# Supplementary material for: Leukemic progenitor compartment serves as a prognostic measure of cancer stemness in patients with acute myeloid leukemia
Source: Cell Rep Med. 2023 Jul 10;4(7):101108. doi: 10.1016/j.xcrm.2023.101108 (PMC10394166; doi:10.1016/j.xcrm.2023.101108)
Supplement: Document S1. Figures S1–S4 and Tables S1–S6 [file mmc1.pdf]

Cell Reports Medicine, Volume *4*

Supplemental information

Leukemic progenitor compartment serves as a prognostic measure of cancer stemness in patients with acute myeloid leukemia

Allison L. Boyd, Justin Lu, Cameron G. Hollands, Lili Alsostovar, Shiva Murali, Jennifer C. Reid, Wendy Ye, Sean Vandersluis, Paige Johnson, Amro ElRafie, Deanna P. Porras, Dimetri Xenocostas, Andrew Leber, Brian Leber, Ronan Foley, Michael Trus, Tobias Berg, Eri Kawata, Anargyros Xenocostas, and Mickie Bhatia

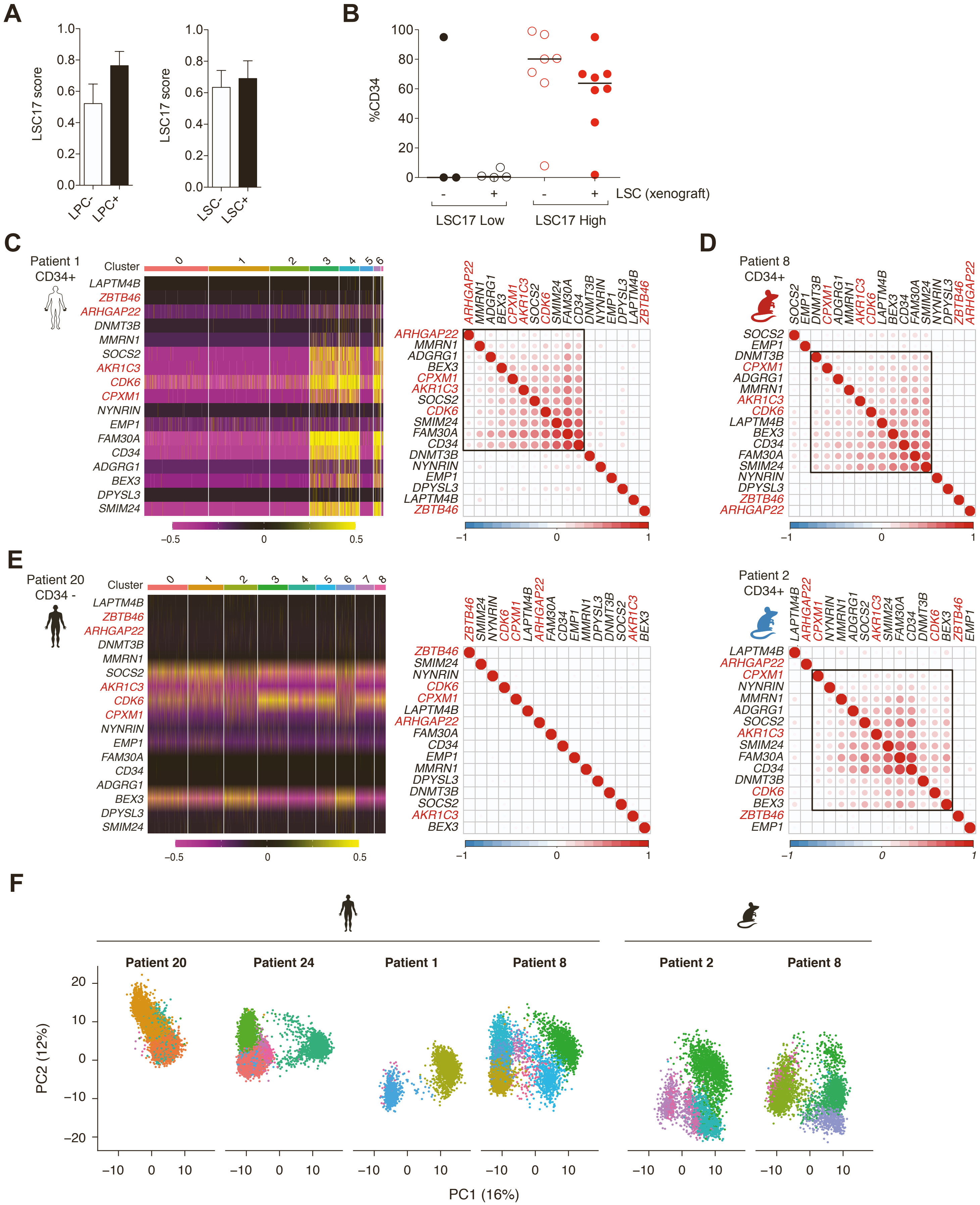

Figure S1. LSC17 scores and single cell LSC17 gene expression in human AML. Related to Figure 1.
(A) Comparison of LSC17 transcriptional scores in LPC- vs. LPC+ or LSC- vs. LSC+ patient subsets (*n*=22 patients total). LSC17 scores were derived from bulk RNA analyzed using the Nanostring platform. (B) Percentage CD34 expression measured by flow cytometry (*n*=22 patients). Black coloring indicates LSC17^{Low} and red coloring indicates LS17^{High}. Solid data points indicate similar classification between LSC17 scores and xenograft assays while hollow data points indicate different classification between LSC17 scores and xenograft assays.

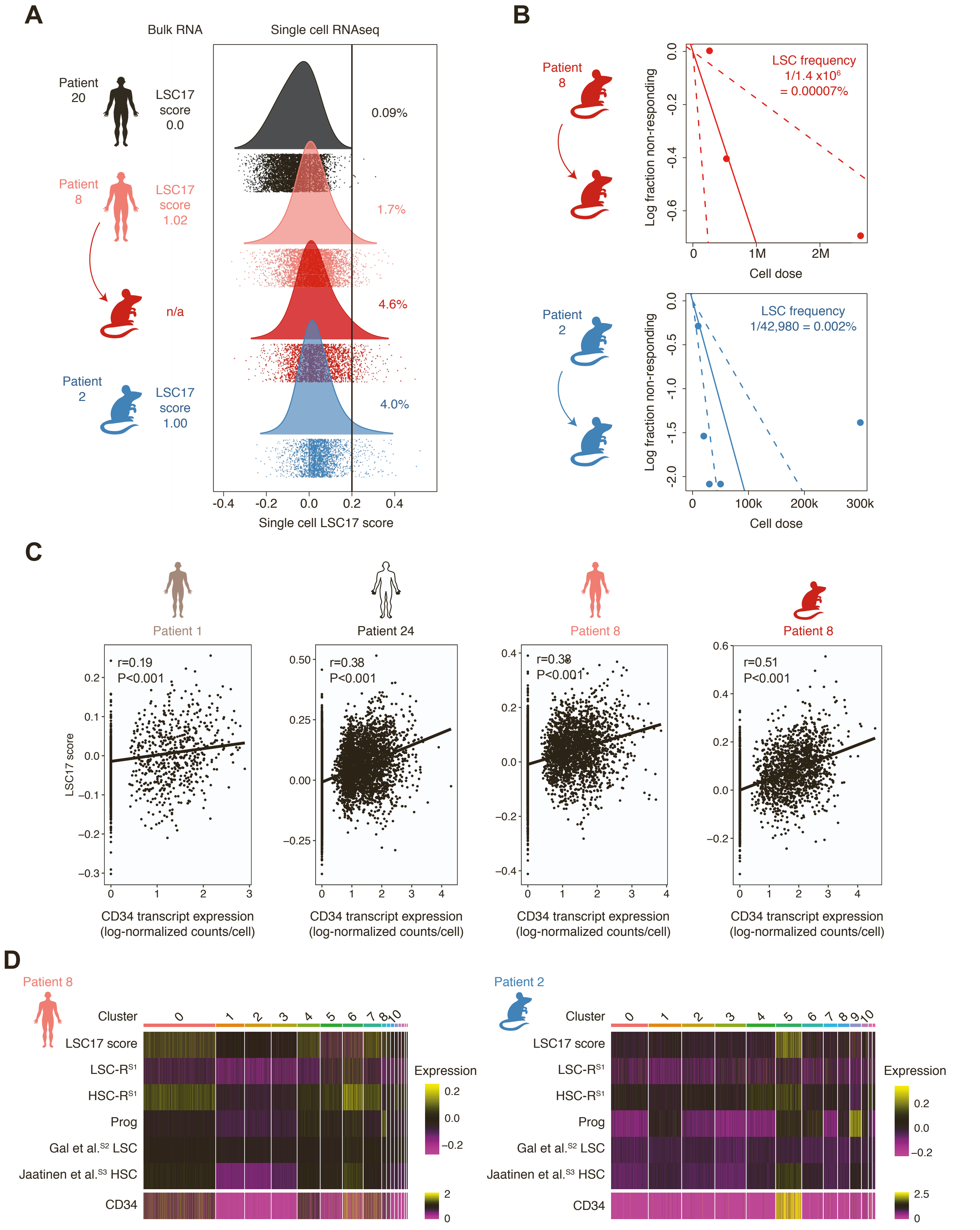

Figure S2. LSC17 scores correlate with CD34 expression at the single cell level. Related to Figure 1.
(A) Histogram showing the distribution of LSC17 transcriptional scores across individual human cells from AML Patient #20 and AML Patient #8, as well as xenografts from AML Patient #8 and AML Patient #2 (FACS-purified for human CD45+CD33+ cells). LSC17 scores derived from bulk RNA are shown for 3 of the samples. Single cell LSC17 score threshold of 0.2 was defined based on the histogram for Patient #20 (LSC17 score = 0 in bulk RNA assay for this patient).

(B) Limiting dilution serial transplantation to quantify functional LSC frequencies in xenografted human cells from AML Patient #2 and AML Patient #8. Both experiments involved IV transplantation of BM cells from 1° NSG recipients into 2° NSG recipients. (C) Correlation between CD34 transcript expression and LSC17 scores in single human AML cells. (D) Heat maps of LSC17 scores and published signatures of LSC/HSC/progenitors in single cells from an AML patient (Patient #8) and purified human cells from an AML-xenograft (Patient #2). LSC-R, HSC-R and Prog (progenitor) signatures are from Eppert et al.[S1], and additional LSC and HSC signatures were obtained from Gal et al.[S2] and Jaatinen et al.[S3], respectively.

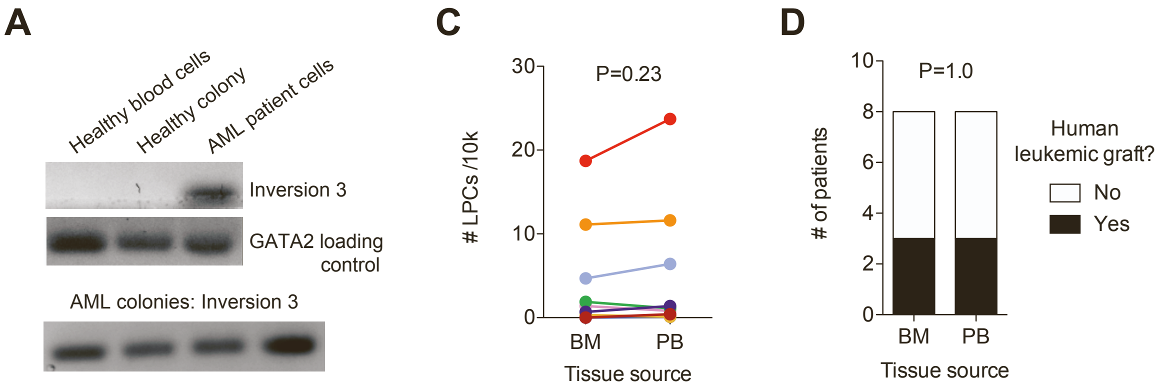

B

Source	# Colonies	Mutation	Detection technique	# Mutant colonies	# Wildtype colonies
Aslostovar et al[S4]	10	-7	ddPCR	10	0
Aslostovar et al[S4]	8	FLT3-ITD	PCR	7	1
Lee et al[S5]	1	MLL-AF9	FISH	25 cells from pooled colonies	0 cells
Patient 20	4	DNMT3A<br>ETV6	ddPCR<br>ddPCR	4<br>3	0<br>1
Patient 105	5	DNMT3A<br>NPM1	ddPCR<br>ddPCR	5<br>5	0<br>0
Patient 8	10	TP53<br>GRM8	ddPCR<br>ddPCR	10<br>10	0<br>0
Patient 106	5	TET2<br>NRAS	ddPCR<br>ddPCR	5<br>5	0<br>0
Patient 107	3	IDH2	ddPCR	3	0
Patient 108	3	ZRSR2	ddPCR	3	0
Patient 109	11	ASXL1	ddPCR	11	0
Patient 2	4	Inversion 3	PCR	4	0
Patient 110	15	STAG2	Sanger	15	0
Totals:	79			78	1 WT, 1 Preleukemic

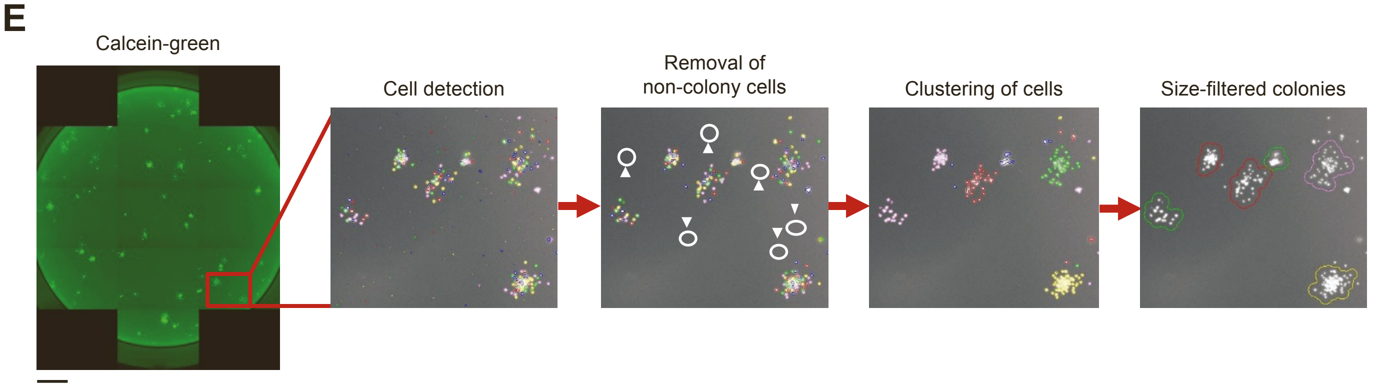

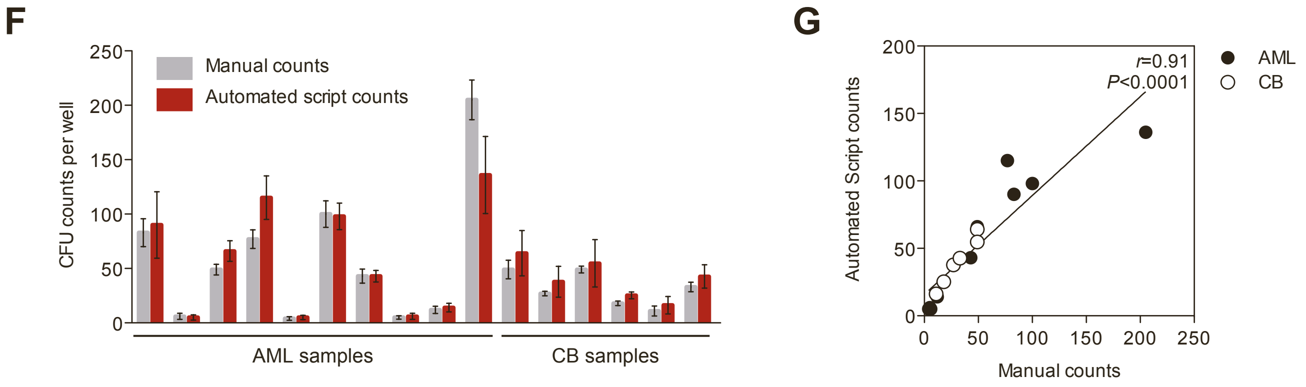

Figure S3. LPC assays are robust, reproducible, and can be automated. Related to Figure 2.
(A) Agarose gel electrophoresis showing PCR product specific for inversion 3 breakpoint region identified for Patient #2. DNA was extracted from bulk mononuclear cells or plucked colonies. (B) Summary of patient-specific mutation detection in plucked colonies, across 12 independent AML patients. For Patient #20, all colonies were counted as mutant due to the detection of at least one patient-specific mutation. (C) LPC frequencies in matched BM and PB samples collected from the same patients (collected <48 hours apart at diagnosis; n=9 patients). (D) Xenograft outcomes in matched BM and PB samples collected from the same patients (collected <48 hours apart at diagnosis; n=8 patients). Xenotransplantation was performed identically between BM and PB for all matched pairs (mouse strain and injection route). (E) Automated colony detection in high-throughput CFU platform. Scale bar, 2mm. (F) Automated script counts vs. manual counts of CFUs (n=3 wells from each of n=10 AML samples and n=5 healthy cord blood samples). CB, healthy cord blood. (G) Correlation of Automated script counts vs. Manual counts of CFUs. CB, healthy cord blood.

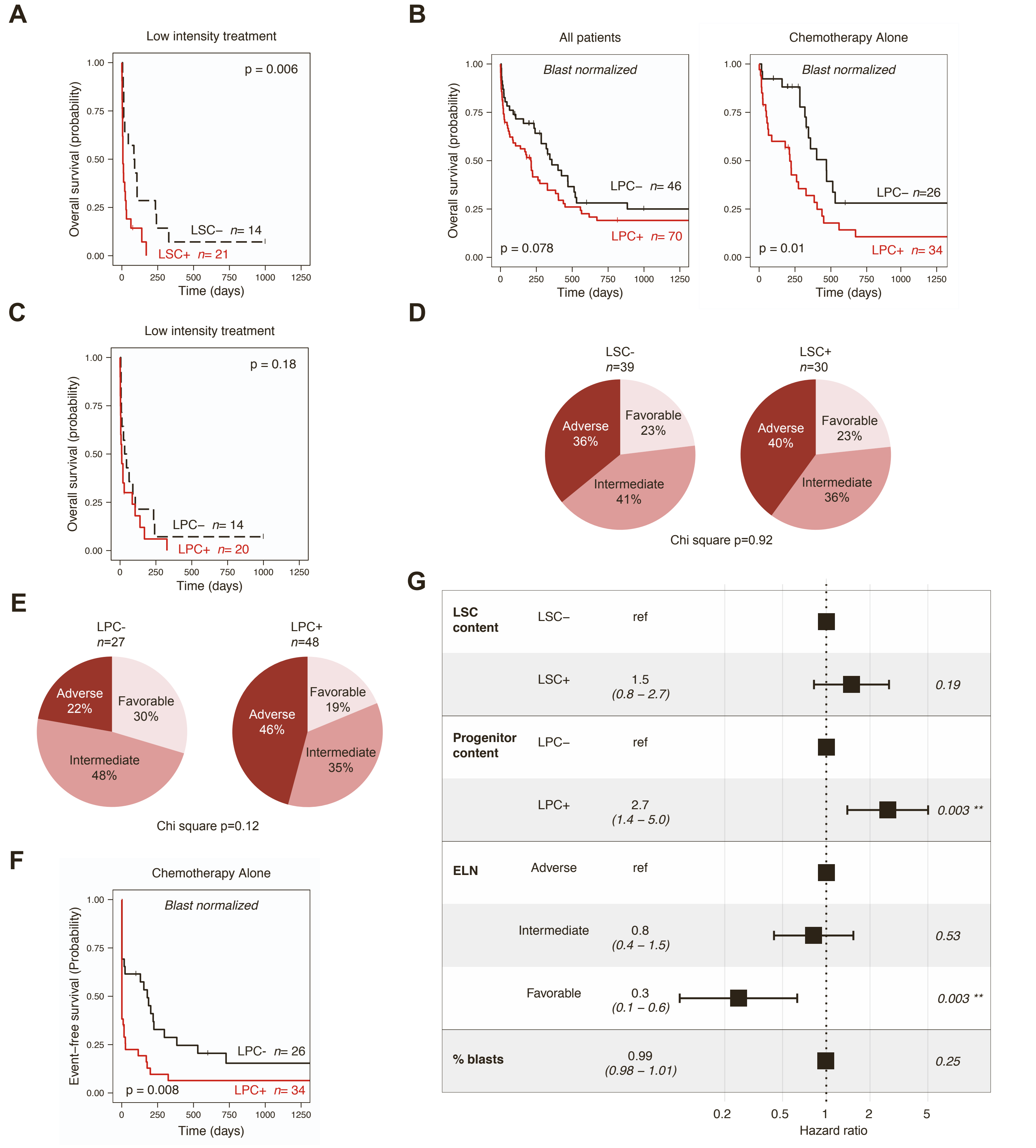

Figure S4. Functional LPC frequencies remain prognostic when normalized for leukemic blast content. Related to Figures 3 and 4.
(A) Kaplan-Meier estimates of Overall Survival in LSC- and LSC+ patient subsets (patients treated with low intensity therapy or palliative care only; *n*=35). (B) Kaplan-Meier estimates of Overall Survival in LPC- and LPC+ patient subsets, defined by LPC frequencies/10k leukemic blasts. Plots display all *n*=116 patients tested in colony forming LPC assays (left), or a subset of these patients treated with high intensity chemotherapy alone (palliative and HSCT-treated patients excluded; right). (C) Kaplan-Meier estimates of Overall Survival in LPC- and LPC+ patient subsets (patients treated with low intensity therapy or palliative care only; *n*=34). (D) Distribution of ELN genetic risk classes in LSC- vs. LSC+ patient subsets (*n*=69). (E) Distribution of ELN genetic risk classes in LPC- vs. LPC+ patient subsets (*n*=75). (F) Kaplan-Meier estimates of Event-free Survival in LPC- and LPC+ patient subsets, defined by LPC frequencies/10k leukemic blasts (patients treated with high intensity chemotherapy alone; *n*=60 patients). (G) Forest plot showing multivariate analysis of Event-free Survival in AML patients treated with high intensity chemotherapy alone (*n*=60 patients). Unadjusted HRs (squares) and 95% confidence intervals (horizontal lines) are shown.

Table S1. Clinical characteristics of patients evaluated by scRNAseq and/or Nanostring assays. Related to Figure 1.

ID#	Age	Sex	Genetics	AML engraftment	LPC freq/ 10k MNC	LSC17 classification	% CD34
1	56	M	46,XY[25]	N/A	N/A	N/A	N/A
2	48	M	45,XY,inv(3)(q21q26.2),-7[25]	Yes	12	High	70.1
3	84	M	Unknown karyotype, FLT3 ITD+NPM1-	No	45	High	7.9
4	71	F	Unknown karyotype, FLT3 ITD-NPM1-	No	0	Low	95
5	79	M	Complex	No	220	High	64.1
6	78	F	Complex	No	0	High	80.2
7	51	F	46,XX [25], FLT3 ITD+NPM1-	Yes	52	High	60
8	78	F	Complex	Yes	12	High	67.6
9	65	F	46,XX[20], FLT3 ITD+NPM1+	Yes	0	High	1.8
10	78	F	Unknown karyotype, FLT3 ITD+NPM1-	No	0	High	99
11	94	M	Unknown karyotype, FLT3 ITD-NPM1-	No	38	High	2
12	65	F	Unknown karyotype, FLT3 ITD+NPM1+	Yes	128	Low	6.8
13	61	M	46,XY,t(4;17)(q21;q21)[23]/46,XY[1], FLT3-ITD+NPM1+	Yes	18	High	59.1
14	56	F	50,XX,+8,+9,+14,+19[21]/46,XX[1]	No	34	Low	0.01
15	67	F	45,XX,-7[19]/46,XX[1]	Yes	45	High	70
16	73	F	Unknown karyotype, FLT3 ITD+NPM1+	Yes	1	Low	0.4
17	67	M	46,XY [3], FLT3 ITD-NPM1-	No	0	High	71.2
18	40	M	46,XY [15], FLT3-ITD+	Yes	28	High	37.3
19	62	M	Unknown karyotype, FLT3 ITD-NPM1+	Yes	0	Low	1
20	42	F	t(9;11), DNMT3A, ETV6, KRAS	Yes	23	Low	0.02
21	28	M	46,XY[20], FLT3 ITD-NPM1+	No	1	Low	0
22	70	F	46,XX [17], FLT3 ITD-NPM1-	Yes	1	High	95
23	65	M	CBFß/MYH11	No	65	High	80.6
24	44	F	46,XX [16], FLT3 ITD+NPM1+	Yes	2	N/A	57.8

Table S2. Hazard Ratios for Overall Survival in a preliminary cohort of 22 AML patients. Related to Figure 1.

Stemness measurement	HR	95% CI
%CD34	1.33	0.49-3.66
LSC17 score	1.39	0.49-3.95
LSC	1.27	0.46-3.55
CFU	7.13	1.55-32.8

Table S3. Serial xenotransplantation of AML patient samples. Related to Figure 1.

	1° xenograft			2° xenograft	
Patient ID	Leukemic chimerism	*n*	Experiment type	Leukemic chimerism	*n*
2	94.2 ± 2.5	5	mouse-to-mouse	94.2 ± 3	5
7	49.9 ± 22.2	4	mouse-to-mouse	64.9 ± 21.9	4
8	77.2 ± 4.6	6	Pooled*	0.4 ± 0.2	6
25	19 ± 4.4	8	Pooled	19.4 ± 3.7	6
26	38.1 ± 4.6	3	Pooled	22.4 ± 1.1	2
27	19.9 ± 8.5	3	Pooled	50.5 ± 8.2	3
28	70.2 ± 5.2	2	mouse-to-mouse	11.4 ±- 6.3	2
29	14 ± 6.9	4	mouse-to-mouse	1.3 ± 0.5	4
30	85.2 ± 6.2	4	mouse-to-mouse	0.1 ± 0.1	3

Chimerism values represent mean ± s.e.m.
*Transplanted at limiting dilution

Table S4. Primer sequences. Related to Figure 2 and STAR Methods.

Target	Application	Forward primer	Reverse primer	Probe 1	Probe 2
DNMT3A c.2645G>A	ddPCR	CCCAGGGTATTTGGTTTCCC	GAAGAGGTGGCGGATGACT	AACATGAGCCACTTG	ACATGAGCCGCTTGG
ETV6 c.331G>A	ddPCR	TGCTGCTCCGTAGATCGTTG	CCGGCTGTGTGTGTATAGAGT	TTCCAGGTAATGTGC	TTCCAGGTGATGTGC
TP53 c.842A>T	ddPCR	GAACAGCTTTGAGGTGCGTG	TGTCCTGCTTGCTTACCTCG	CCTGGGAGAGTCC	TCCTGGGAGAGACC
GRM8 c.10G>A	ddPCR	GTAGCCTCCAGAAGGTGCAG	CCAGTAGAACTTGGCGGTCA	TGGTATGCAAGGGAA	TGGTATGCGAGGGAA
NRAS c.38G>T	ddPCR	ACAGGTTCTTGCTGGTGTGAA	TGGATTGTCAGTGCGCTTTTC	AGCAGGTG**G**TGTTGG	AGCAGGTG**T**TGTTGG
TET2 c.1630C>T	ddPCR	TGCCAGCAGTTGATGAGAAACA	TCCGCTTGGTGAAAACGAGG	ATTCTGAAGGGT**C**GAGAC	ATTCTGAAGGGT**T**GAGAC
IDH2 c.419G>A	ddPCR	TGGGCTCCCGGAAGACA	AAGATGTGGAAAAGTCCCAATGGA	CTATCCGGAACATCC	ACTATCCAGAACATCC
ZRSR2 c.810_811insAT	ddPCR	GTCAGCTGCAATTTGGAACCT	GGAAGACATCCACAAGCAGAATACT	CTGGTACTGAACATATACATT	TGGTACTGAACATAATTACATT
ASXL1 c.2725A>T	ddPCR	CGTTTCTAACAGTTCTTTGCATTGGA	TGTGTTCTCTGGATTCTGGTTTGG	TGAGGTAGTGAAACAGC	ATGAGGTAGTGTAACAGC
STAG2:c.2924+3A>C	conventional PCR	GCTCGACGTTTTGCTTTAACT	AGATAGCACTGTAACTGGTTCTT	N/A	N/A
Inversion 3	conventional PCR	CCTCTGAGCAACTTTGCTTTC	GGCTGGCAGAAAATCTTCAC	N/A	N/A

Table S5. Multivariate analysis of Overall Survival in AML patients treated with high intensity chemotherapy alone (*n*=60 patients), classified based on ELN 2022 risk status. Related to Figure 4.

Variable		Hazard ratio	P value
LSC content	LSC-	reference	
	LSC+	0.98 (0.49 - 1.96)	0.96
LPC content	LPC-	reference	
	LPC+	2.63 (1.21 - 5.72)	0.015*
ELN 2022	Adverse	reference	
	Intermediate	1.45 (0.70 - 2.99)	0.31
	Favorable	0.28 (0.08 - 0.97)	0.04*
% blasts		1.02 (0.995 - 1.05)	0.1

*statistically significant

Table S6. Multivariate analysis of Event-free Survival in AML patients treated with high intensity chemotherapy alone (*n*=60 patients), classified based on ELN 2022 risk status. Related to Figure 4.

Variable		Hazard ratio	P value
LSC content	LSC-	reference	
	LSC+	1.52 (0.81 - 2.83)	0.19
LPC content	LPC-	reference	
	LPC+	2.67 (1.29 - 5.49)	0.008*
ELN 2022	Adverse	reference	
	Intermediate	1.02 (0.49 - 2.08)	0.97
	Favorable	0.22 (0.08 - 0.64)	0.006*
% blasts		0.99 (0.98 - 1.01)	0.26

*statistically significant

Supplemental References

S1. Eppert K, Takenaka K, Lechman ER, Waldron L, Nilsson B, van Galen P, Metzeler KH, Poeppl A, Ling V, Beyene J, et al. (2011). Stem cell gene expression programs influence clinical outcome in human leukemia. Nat Med 17, 1086-1093. 20110828.

S2. Gal H, Amariglio N, Trakhtenbrot L, Jacob-Hirsh J, Margalit O, Avigdor A, Nagler A, Tavor S, Ein-Dor L, Lapidot T, et al. (2006). Gene expression profiles of AML derived stem cells; similarity to hematopoietic stem cells. Leukemia 20, 2147-2154. 20061012.

S3. Jaatinen T, Hemmoranta H, Hautaniemi S, Niemi J, Nicorici D, Laine J, Yli-Harja O and Partanen J. (2006). Global gene expression profile of human cord blood-derived CD133+ cells. Stem Cells 24, 631-641. 20051006.

S4. Aslostovar L, Boyd AL, Benoit YD, Di Lu J, Garcia Rodriguez JL, Nakanishi M, Porras DP, Reid JC, Mitchell RR, Leber B, et al. (2021). Abnormal dopamine receptor signaling allows selective therapeutic targeting of neoplastic progenitors in AML patients. Cell Rep Med 2, 100202. 20210216.

S5. Lee JH, Salci KR, Reid JC, Orlando L, Tanasijevic B, Shapovalova Z and Bhatia M. (2017). Brief Report: Human Acute Myeloid Leukemia Reprogramming to Pluripotency Is a Rare Event and Selects for Patient Hematopoietic Cells Devoid of Leukemic Mutations. Stem Cells 35, 2095-2102. 20170731.
