# Supplementary material for: Leukemic progenitor compartment serves as a prognostic measure of cancer stemness in patients with acute myeloid leukemia
Source: Cell Rep Med. 2023 Jul 10;4(7):101108. doi: 10.1016/j.xcrm.2023.101108 (PMC10394166; doi:10.1016/j.xcrm.2023.101108)
Supplement: Document S2. Article plus supplemental information [file mmc2.pdf]

Cell Reports
Medicine

Report

Leukemic progenitor compartment serves as a prognostic measure of cancer stemness in patients with acute myeloid leukemia

Graphical abstract

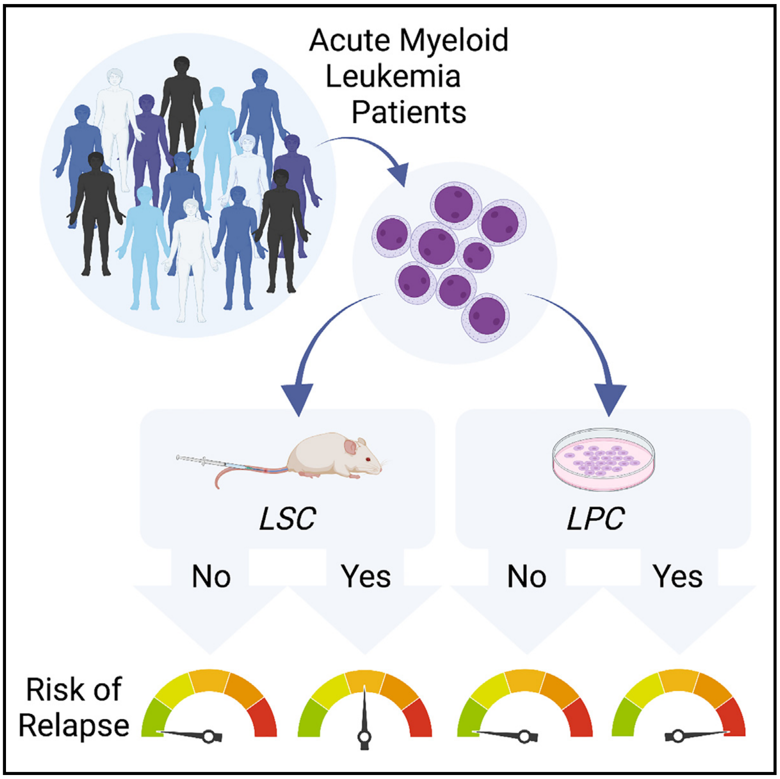

Highlights

- Comparative study of features of cancer stemness in a single patient cohort
- Leukemic progenitor cells (LPCs) capture patient specific mutations
- LPCs provide a robust prognostic predictor of survival in patients with AML
- Prognostic value of LPCs is independent of current genetic risk classification

Authors

Allison L. Boyd, Justin Lu, Cameron G. Hollands, ..., Eri Kawata, Anargyros Xenocostas, Mickie Bhatia

Correspondence

mbhatia@mcmaster.ca

In brief

Boyd et al. compare measures of stemness in patients with acute myeloid leukemia (AML). While the presence of xenograft-detected leukemic cells predicts poor survival, *in vitro* colony formation reveals that leukemic progenitor cells (LPCs) more strongly correlate with overall and event-free survival. LPCs provide a rapid and robust functional measure of AML disease.

Boyd et al., 2023, Cell Reports Medicine *4*, 101108
July 18, 2023 Crown Copyright © 2023
https://doi.org/10.1016/j.xcrm.2023.101108

Cell Reports Medicine

CellPress OPEN ACCESS

Report

Leukemic progenitor compartment serves as a prognostic measure of cancer stemness in patients with acute myeloid leukemia

Allison L. Boyd,[1] Justin Lu,[1] Cameron G. Hollands,[1] Lili Alsostovar,[1] Shiva Murali,[1] Jennifer C. Reid,[1] Wendy Ye,[1] Sean Vandersluis,[1] Paige Johnson,[1] Amro ElRafie,[1] Deanna P. Porras,[1] Dimetri Xenocostas,[1] Andrew Leber,[1] Brian Leber,[2] Ronan Foley,[3] Michael Trus,[2] Tobias Berg,[3] Eri Kawata,[4] Anargyros Xenocostas,[4] and Mickie Bhatia[1,5,*]

[1]Michael G. DeGroote School of Medicine, Department of Biochemistry and Biomedical Sciences, McMaster University, Hamilton, ON L8N 3Z5, Canada

[2]Department of Pathology and Molecular Medicine, McMaster University, Hamilton, ON L8S 4L8, Canada

[3]Department of Oncology, McMaster University, Hamilton, ON L8S 4L8, Canada

[4]Department of Medicine, Division of Hematology, Schulich School of Medicine, University of Western Ontario, London, Ontario N6A 3K7, Canada

[5]Lead contact

*Correspondence: mbhatia@mcmaster.ca

https://doi.org/10.1016/j.xcrm.2023.101108

SUMMARY

We systematically investigate functional and molecular measures of stemness in patients with acute myeloid leukemia (AML) using a cohort of 121 individuals. We confirm that the presence of leukemic stem cells (LSCs) detected through *in vivo* xenograft transplantation is associated with poor survival. However, the measurement of leukemic progenitor cells (LPCs) through *in vitro* colony-forming assays provides an even stronger predictor of overall and event-free survival. LPCs not only capture patient-specific mutations but also retain serial re-plating ability, demonstrating their biological relevance. Notably, LPC content represents an independent prognostic factor in multivariate analyses including clinical guidelines of risk stratification. Our findings suggest that LPCs provide a robust functional measure of AML, enabling quantitative and rapid assessment of a wide range of patients. This highlights the potential of LPCs as a valuable prognostic factor in AML management.

INTRODUCTION

Poor survival rates among patients with acute myeloid leukemia (AML) have been attributed to the persistence of therapy-resistant cancer stem cells (CSCs). In AML, however, the majority of patients lack bona fide leukemic stem cells (LSCs) defined by the ability to generate patient-specific leukemia in human-mouse xenografts upon *in vivo* transplantation.[1,2] Despite the finding that patients' leukemic cells that possess LSC capacity by xenograft detection (LSC+ patients) have worse prognoses than patients lacking detectable LSCs (LSC− patients),[1,3,4] these assays are laborious and cost prohibitive, which makes their application impractical for many researchers and also for clinical settings.

Original qualitative descriptions of CSCs in AML suggested that they resided exclusively within the $CD34^{+}CD38^{-}$ phenotypic fraction of AML patient cells.[5,6] Based on this view, multiple groups have explored the clinical significance of these phenotypes and have reported relationships between traditional $CD34^{+}CD38^{-}$ profiles and AML patient survival.[7,8] However, collective studies indicated that LSC immunophenotypes are considerably heterogeneous across patients, as LSCs are often also found in $CD34^{+}CD38^{+}$ fractions and occasionally also in subsets lacking CD34 expression.[9–11] Furthermore, the labor, expense, and expertise required for *in vivo* transplantation assessment of CSCs and the need to collect large numbers of viable cells from patients collectively limit the direct utility of xenograft assays for routine clinical decision-making and management of patients with cancer. To address phenotypic inconsistencies and practical complications of *in vivo* LSC detection, a transcriptional signature for AML-LSCs was defined by fractionating patient cells into defined subsets and testing each fraction for functional LSC potential.[9] The resulting signature represented functional engrafting vs. non-engrafting subsets and was found to correlate with patient survival.[9] More recent efforts to continue to capitalize on the prognostic value of complex LSC measurements has led to the development of a prognostic "LSC17" gene expression score to be generated for patients with AML[12,13] by statistical training using patient survival data.

Alternatively, assays to interrogate stemness using less laborious approaches that require small numbers of patients' cells have been investigated and include *in vitro* assays such as AML cell coculture with bone marrow (BM) stroma in long-term culture-initiating cell assays or direct semi-solid colony-forming

This is an open access article under the CC BY license (http://creativecommons.org/licenses/by/4.0/).

unit (CFU) assays.[14,15] Over two decades ago, leukemic cells detected in these *in vitro* systems have allowed clonogenic self-renewal capacity to be measured more quantitatively than xenograft-defined LSCs and have also been suggested to predict AML patient survival.[15–18] However, currently evolved molecular risk stratification methods could not be applied in these historical studies, and it remains unknown whether leukemic progenitors would add prognostic value beyond contemporary European Leukemia Net risk assessment for AML. In addition, *in vitro* approaches have not been directly compared, on a patient-by-patient basis, with other stemness-based measurements including *in vivo* LSC detection or LSC transcriptional scores. Here, our results from a systematic and comparative study of features of cancer stemness reveal variability among different assays and suggest that leukemic progenitor cell (LPC) frequencies offer an alternative functional measure of cancer stemness that is both clinically relevant and accessible to a wide range of researchers.

RESULTS

Independent phenotypic, molecular, and functional measurements of leukemic stemness are slightly variable in cohort of patients with AML

Although the field has invested considerable effort to develop different functional and molecular measures to estimate features of leukemic stemness in samples obtained from patients with AML, few studies have comprehensively compared different measurements across the same set of patients.[19] Using a heterogeneous group of 22 patients with AML (patients #2–23; Table S1), we first prioritized functional biological testing to identify LSCs and LPCs. Our functional analyses consisted of a series of *in vivo* and *in vitro* assays using xenograft modeling to rigorously detect LSCs, as well as semi-solid CFU assays to detect LPCs (Figure 1A). Patient samples were selected for molecular profiling to deliberately represent all four outcomes of our functional assays (i.e., LSC+LPC+, LSC+LPC−, LSC−LPC−, LSC−LPC+). We then further investigated how these functional measures compare with recently established LSC17 scores,[12] as well as CD34 cell surface protein expression. The LSC17 score represents a clinical-grade gene expression assay that has been widely validated to predict AML patient survival by multiple independent groups.[12,13] This score was originally established by comparative gene expression of sorted AML cell fractions that contained functionally detectable LSCs vs. those that lacked functional LSCs, and the genes identified were further trained against AML patient survival outcomes and refined to generate an LSC17 score.[9] Despite the initial development of this score based on the ability of AML cell fractions to engraft mice, it has not yet been revisited whether LSC17 scores correlate with the functional properties of unfractionated samples.

In our cohort of 22 patients, LSC17 scores were slightly higher in LPC+ vs. LPC− groups, but this was not significant (Figure S1A). This was not surprising, as the genes in the LSC17 score were based on xenograft (LSC) assays, and findings from the *in vitro* LPC assay do not always correspond with the *in vivo* LSC assay (Figure 1A). LSC17 scores were also not significantly different between LSC+ vs. LSC− groups, as only 11/22 patients were categorized similarly between the functional LSC assay vs. the molecular LSC17 score (i.e., $LSC^{+}LSC17^{High}$ or $LSC^{-}LSC17^{Low}$; Figures S1A and S1B). While technical barriers associated with xenotransplantation provide a possible explanation for the observation of $LSC^{-}LSC17^{High}$ samples, it was more surprising that over half of the $LSC17^{Low}$ samples produced positive leukemic engraftment in the xenograft assay (4 patients; Figures 1A and S1B). All 4 of these patients lacked expression of the cell surface marker CD34, which has often been associated with functional LSC activity[9] (Figure S1B). The high proportion of $CD34^{Low}$ AML cases in our study (Table S1) represents a possible difference from the patient cohort used to initially derive the LSC17 score. In fact, LSC17 scores strongly correlated with CD34 levels across the full 22-patient cohort (Figure 1B), suggesting that the genes used to calculate the LSC17 score could be more strongly associated with $CD34^{+}$ LSCs vs. $CD34^{-}$ LSCs.

We further investigated this relationship between CD34 and LSC17 scores at the single-cell level by examining cells obtained *de novo* from human patients with AML as well as purified human cells from patient-derived xenografts that were confirmed to self-renew by serial transplantation (a total of 6 samples from 5 patients; Figures S1C–S1E and Figure S2A–S2C). Across cells, the expression levels of most LSC17 genes were positively correlated with each other in AML samples that expressed CD34 (Figures S1C–S1D), but the 17 genes were not correlated in the patient sample that lacked CD34 expression (Figure S1E). The lack of correlation was not due to greater diversity of leukemic cell types within the $CD34^{-}$ sample, as this sample was one of the most cellularly homogeneous of all samples examined (patient #20; Figure S1F). We next calculated LSC17 scores for individual cells, where we found more $LSC17^{High}$ cells in leukemic samples that also had high LSC17 scores by NanoString analysis (i.e., bulk RNA; Figure S2A). Finally, we found that CD34 RNA expression positively correlated with single-cell LSC17 scores across all $CD34^{+}$ samples (Figures 1C, 1D, and S2C), consistent with our observations from bulk RNA analysis (Figure 1B). This is a logical finding given that CD34 is one of the 17 genes and contributes positively to the LSC17 score. In addition to correlation with CD34 expression, we found that there tended to be high LSC17 scores in cell populations enriched for healthy HSC signatures[9,20] (Figure S2D). At the single-cell level, LSC17 scores were less correlative with published LSC signatures, whether phenotypically defined[21] or not[9] (Figure S2D). While the LSC17 score is a robust prognostic tool that was not intentionally developed to act as a molecular "detector" of LSCs, overall, our findings suggest that the genes used to generate this score are predominantly found within $CD34^{+}$ fractions (Figures 1B–1E, S2C, and S2D). This provides some context to understand cases where low LSC17 scores are seen in patient samples that contain both functional LSCs and LPCs but have $CD34^{-}$ phenotypes (e.g., patient with AML #20).

Having established that there is a lack of redundancy between different assays for AML stemness, we explored the clinical relevance of each measure comparatively. Of all four parameters, LPC frequencies showed the strongest relationship with overall survival in this cohort of 22 patients (Figure 1E; Table S2). This smaller cohort may not be sufficiently powered for significant

CellPress
OPEN ACCESS

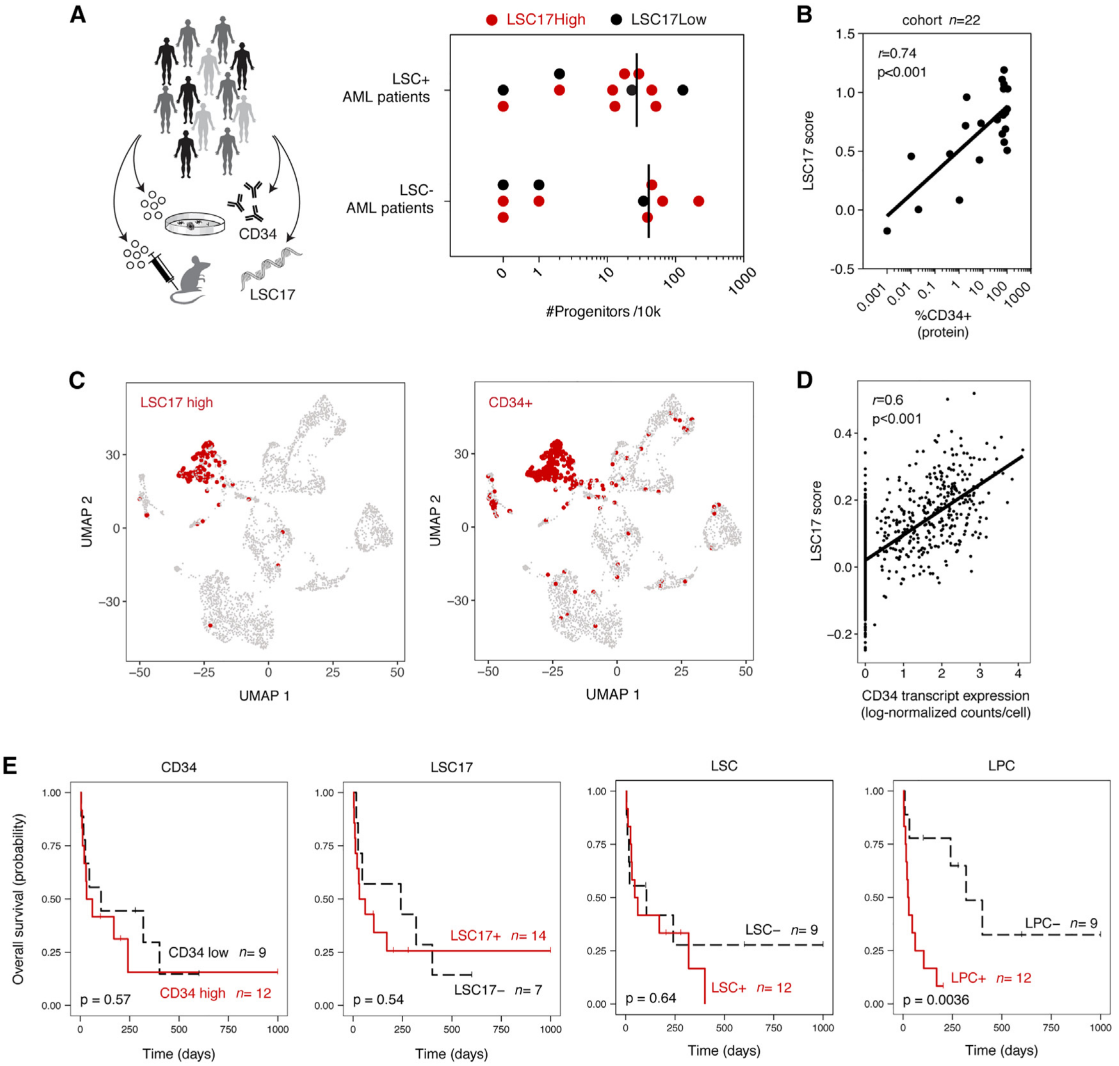

Figure 1. Substantial variation exists between different functional and molecular measures of leukemic stemness

(A) Relationships between engraftment ability, progenitor frequencies, and LSC17 scores (NanoString assay) in a subset of n = 22 patients with AML. Vertical lines indicate the mean LPC frequency for each group.

(B) Correlation between CD34 expression by flow cytometry and LSC17 scores by NanoString across a subset of n = 22 patients with AML.

(C) Uniform manifold approximation and projection (UMAP) dimensionality reduction plots showing gene expression profiles of individual leukemic cells from a human patient with AML (patient #2, xenografted cells that were fluorescence-activated cell sorting (FACS) purified using human markers). Red color indicates single-cell LSC17 score >0.2 (left) or CD34 transcript expression >0 (right). The threshold of 0.2 for the LSC17 score was defined based on Figure S2A.

(D) Correlation between CD34 transcript expression and LSC17 scores in single human AML cells (patient #2, xenografted cells that were FACS purified using human markers).

(E) Kaplan-Meier estimates of overall survival based on CD34 expression (flow cytometry), LSC17 score (NanoString assay), LSC content (xenograft assay), or LPC content (CFU assay). n = 21 patients with AML.

$p < 0.05$ is considered statistically significant.

See also Figures S1 and S2 and Tables S1–S3.

prognostic effects of LSC17 scores or functionally defined LSCs *in vivo* to be observed, as both may require larger datasets to be associated with AML patient survival outcomes.[12,13] Accordingly, we further examined the diagnostic performance of each measurement by evaluating their abilities to predict patient survival at 6 months. In this case, both the LSC17 signature and the LPC assay had superior sensitivity than the traditional LSC xenograft assay or CD34 quantification measured by flow cytometry (Table 1). This could be a reflection of technical challenges associated with LSC detection in xenograft models. Furthermore, despite close correspondence between CD34 and LSC17 scores (Figures 1B–1E, S2C, and S2D), the LSC17 signature provides superior discriminatory power to predict patient outcomes vs. CD34 alone. This reinforces the prognostic value of this clinically developed test. Finally, LPC detection possessed the greatest specificity across all four comparative

Table 1. Diagnostic performance of molecular and functional stemness measurements

	CD34	LSC17	LSCs	LPCs
Sensitivity (%)	61.5 (31.6–86.1)	76.9 (46.2–95.0)	61.5 (31.5–86.1)	84.6 (54.6–98.1)
Specificity (%)	57.1 (18.4–90.1)	57.1 (18.4–90.1)	42.9 (9.9–81.6)	85.7 (42.1–99.6)
Positive predictive value (%)	72.7 (50.6–87.4)	76.9 (57.4–89.2)	66.7 (48.0–84.6)	91.7 (63.8–98.6)
Negative predictive value (%)	44.4 (23.8–67.2)	57.1 (29.0–81.3)	37.5 (16.7–64.3)	75.0 (44.7–91.8)
Positive likelihood ratio	1.44 (0.55–3.74)	1.79 (0.73–4.44)	1.08 (0.50–2.33)	5.92 (0.95–36.9)
Negative likelihood ratio	0.67 (0.26–1.72)	0.4 (0.12–1.32)	0.90 (0.30–2.69)	0.18 (0.05–0.67)

measurements (Table 1). Test specificity is particularly important for clinical decision-making when considering aggressive interventions such as BM transplantation that carry considerable treatment-related mortality and morbidity.

Although the development of xenograft assays, LSC17 scores, and LPC assays have all been motivated by a common effort to understand properties of leukemic stemness, our comparative analysis has suggested that they are not redundant measures. While the xenograft model and the LSC17 score have already been well established to have clear clinical relevance by multiple independent groups,[12,13] LPC assays have received comparatively less attention in this context. Relative to xenografts, LPC assays are accessible to a wider range of research groups, as they can be carried out in standard tissue culture facilities and consume fewer resources. Therefore, we chose to pursue a more comprehensive study of the biological and clinical utility of this assay.

Leukemic progenitor assays capture patient-specific features of disease

Given the promising clinical predictive value of the LPC assay, we invested in further characterizing its biological suitability as a disease model using standards frequently applied to xenograft assays as a benchmark. Human cell outgrowth in patient-derived xenografts is considered to be leukemic when grafts are fully composed of myeloid cells without evidence of balanced multilineage reconstitution, and this practice has been validated through genetic characterization of purified xenografted cells.[2] In LPC assays, the leukemic nature of colonies is distinguishable by their abnormal morphological appearance, as AML patient cells produce irregular myeloid colonies that typically contain fewer cells than healthy myeloid CFU-G (granulocyte) or CFU-M (macrophage) progenitors.[22] There is also little colony-to-colony variation in the morphology of AML-CFUs,[22] which is unlike healthy progenitor cell cultures, where a diversity of different colony types is seen.[22] In one patient case (patient with AML #20), we observed a rare instance of multiple distinct colony types where abnormal myeloid colonies outnumbered scarce erythroid colonies by a frequency of 150:1 (Figures 2A–2C). For this patient, DNA sequencing had revealed multiple high-frequency mutations in known driver genes, including a DNMT3A R88H mutation (c.2645G>A) and a protein-coding mutation in the ETV6 gene. This provided an opportunity to explore whether morphologically different colonies reflect distinct genetics, as DNMT3A mutations can often be pre-leukemic events.[23] Colonies were individually plucked from the culture, and the presence of each mutation was tested using tailored droplet digital PCR (ddPCR) assays. Remarkably, all of the myeloid colonies were mutant for both DNMT3A and ETV6, while the erythroid colony only contained the DNMT3A mutation and was wild type for ETV6 (Figure 2D). This indicates that the LPC assay has the sensitivity to detect both leukemic and pre-leukemic clones, but the outgrowth of pre-leukemic clones is a very infrequent event.

We then expanded our genetic characterization of AML-derived colonies to include a wider range of patients, with an emphasis on maximizing the diversity of different mutations examined. We prioritized patients with known somatic nucleotide variants in driver genes at an estimated cellular prevalence >80% in the *de novo* patient sample (i.e., a variant allele frequency >40% in a heterozygous state). This was to avoid reliance on lower-prevalence mutations that define subclones but may not allow us to confidently distinguish leukemic vs. non-leukemic colonies. Using ddPCR, we evaluated 8 different mutations across 36 colonies and uniformly detected the patient-specific mutation(s) in all cases (Figure 2E). In our broader experience including colony evaluation by fluorescence *in situ* hybridization (FISH) and conventional PCR,[22,24] we have only failed to confirm the presence of a leukemic or pre-leukemic mutation in 1 colony out of 79 (12 patients in total), and in this case, the mutation probed was a FLT3 internal tandem duplication (ITD) that may have been subclonal (Figures S3A and S3B). Collectively, our findings indicate that colony outgrowth from AML patient samples is reliably leukemic in origin with minimal contribution from healthy contaminant.

A second hallmark of LSC assays is the demonstration of self-renewal by serial xenotransplantation. Altogether, we have performed rigorous serial transplantation experiments involving 9 diverse patients, 3 of whom were part of our original 22-patient cohort (Table S3). We detected positive evidence of leukemic self-renewal in all 9/9 cases, although secondary engraftment levels were highly variable across patient samples. *In vitro*, we have similarly performed serial re-plating assays to evaluate the ability of leukemic progenitors to form secondary colonies upon repeated functional challenge (Figure 2F). In some cases, colony-forming ability markedly increased from primary to secondary wells, whereas in other cases it remained stable or decreased (Figure 2G). This pattern resembled our findings in secondary transplantation experiments *in vivo* (Table S3). Finally, we considered whether the tissue source of the leukemic blasts influences the functional measurement of LPCs. For a subset of 12 patients, we received paired peripheral blood (PB) and BM samples collected no more than 48 h apart, prior to the initiation of induction chemotherapy treatment. *In vitro* CFU assays were

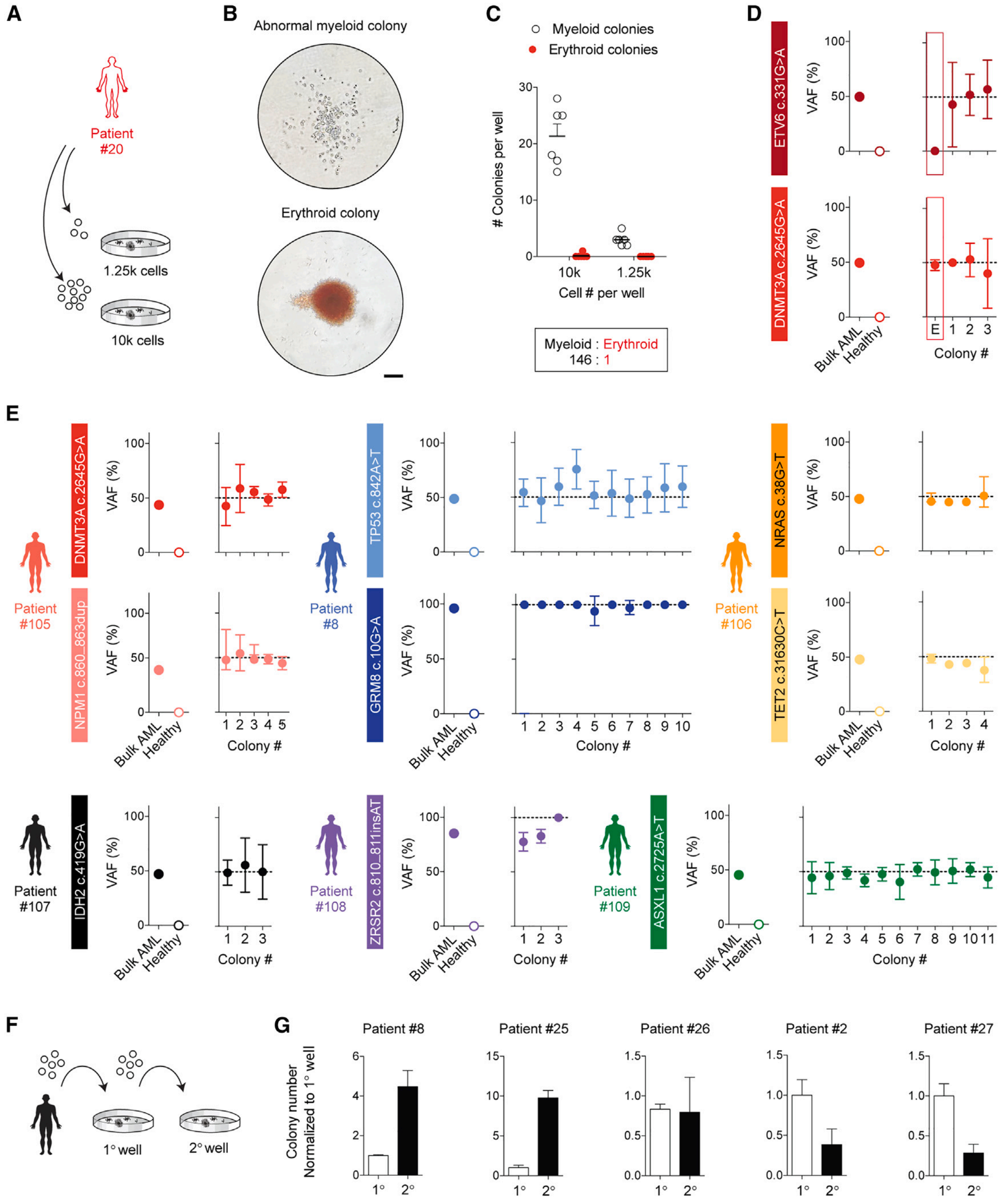

Figure 2. Leukemic progenitor assays capture patient-specific mutations and serial re-plating ability

(A) Schematic outlining LPC assays performed with cells from patient #20 (results shown in B–D). Following 14 days of incubation, colonies were imaged, quantified, and plucked for DNA extraction.

(B) Representative bright-field images of abnormal myeloid colonies and erythroid colonies from patient with AML #20 (scale bar, 100 μm).

(C) Colony counts from LPC assays with cells from patient #20 (counts from n = 12 wells, n = 1 patient). Data are shown as mean ± SEM.

(D) The variant allele frequency (VAF) of patient-specific mutations measured in DNA extracted from bulk mononuclear cells or plucked colonies, measured by ddPCR (patient #20). E, erythroid colony. Red rectangles highlight the colony that was mutant for only one of two patient-specific mutations tested. Healthy indicates healthy umbilical cord blood cells (n = 4 colonies from n = 1 patient). Error bars represent the upper and lower limits of the Poisson fractional abundance.

(E) The VAF of patient-specific mutations measured in DNA extracted from bulk mononuclear cells or plucked colonies, measured by ddPCR (n = 36 colonies from n = 6 patients). Patient IDs are indicated next to graphs. Healthy indicates healthy umbilical cord blood cells. Error bars represent the upper and lower limits of the Poisson fractional abundance.

(F) Schematic showing the collection of entire 1° CFU wells to be plated into 2° CFU wells to measure the serial re-plating ability of LPCs.

(legend continued on next page)

performed using paired BM and PB samples from 9 of these patients, and we did not detect a significant difference in LPC frequencies between the two tissue sources (Figure S3C). *In vivo* xenotransplantation assays were performed with paired samples from 8 of these patients, again with the same outcomes between BM and PB (Figure S3D). This matches previous reports by others who have also concluded that BM and PB sources of leukemic blasts are functionally similar.[1]

Upon establishing the biological robustness of progenitor assays for human AML, we adapted a high-throughput leukemic CFU platform for efficient quantification of leukemic progenitors *in vitro*. The workflow was streamlined to standardize colony quantification via high content imaging techniques and customized image analysis scripting (Figure S3E). This computational approach was tested against manual scoring of progenitor colonies and was shown to match (Figures S3F and S3G), thereby confirming the reliability of our automated approach that allowed us to examine larger cohorts of patients with AML in a non-biased manner.

LPC content emerges as a robust predictor of AML patient outcome

Findings from our initial 22-patient cohort underscored the potential value of LPC detection as a prognostic factor and motivated us to further evaluate a larger set of patients and additionally consider whether prognostic impacts of stemness measures are independent of conventional risk assessment standards for AML. Accordingly, we expanded our clinical study to 121 patients with AML treated at two separate sites. Detailed AML patient characteristics are shown Table 2, with an estimated median follow-up time of 1,757 days (95% confidence interval [CI], 1,608–2,583 days). In parallel with our LPC assays in this wider group, we also continued to perform LSC testing in xenograft models for the vast majority of patient samples. A total of 111/121 patients were tested for LSC content in immune-deficient xenograft models, exceeding previous studies for functional *in vivo* LSC evaluation that ranged from 25 to 56 patients.[1,3,4,25] When using this larger patient dataset, and consistent with previous reports,[1,3,4,25] patients with detectable LSCs had shorter overall survival (OS) than those without LSCs (Figure 3A; hazard ratio [HR] 1.81; 95% CI, 1.17–2.82). While previous studies are mixed with regard to the inclusion of patients treated palliatively[3] vs. strict evaluation of patients treated with standard chemotherapy regimens alone,[1] assessments of risk biomarkers should be considered in the context of therapeutic treatment. Our full patient cohort includes patients treated palliatively, patients treated with standard chemotherapy alone, and patients treated with standard chemotherapy plus hematopoietic stem cell transplantation (HSCT). When we examined a more uniform subgroup of patients that were specifically treated with high-dose chemotherapy alone, the presence of LSCs was still negatively associated with OS; however, this was no longer statistically significant (Figure 3A; HR 1.79; 95% CI, 0.93–3.46, p = 0.077). Surprisingly, LSCs were negatively associated with OS in patients who received low-intensity treatment (e.g., palliative care; Figure S4A; HR 2.88; 95% CI, 1.32–6.29, p = 0.008), suggesting that there is a relationship between LSCs and survival even in the absence of chemotherapy treatment. Given that HSCT represents a highly prioritized therapy for patients who are suitable candidates, we further explored the prognostic significance of LSCs in the context of therapy treatment by additionally considering patients treated with chemotherapy who also received allogeneic HSCT (n = 23 patients). When HSCT was included as a time-dependent covariate, LSC content no longer maintained a significant impact on OS (Figure 3B; HR 1.34; 95% CI, 1.14–1.54, p = 0.18), and HSCT did not have a significant impact either (Figure 3B; HR 0.86; 95% CI, 0.59–1.13, p = 0.35). Overall, our wider results (Figure 3A) correspond with those of several independent groups that converge to suggest that functionally defined LSCs are a negative prognostic factor in adult AML.[1,3,4,25]

A total of 116 patients were evaluated for leukemic progenitor content, mostly overlapping with the patients tested for LSC content in xenograft assays. As we observed with the *in vivo* LSC detection, OS was shorter for patients with abundant leukemic progenitors (Figure 3C; HR 1.73; 95% CI, 1.11–2.71), and this effect was more pronounced in patients treated with standard induction chemotherapy alone (Figure 3C; HR 3.29; 95% CI, 1.71–6.34). This remained true when LPC frequencies were calculated as a proportion of total leukemic blasts rather than LPC as a proportion of total cells (Figure S4B; HR 1.89; CI, 1.2–3.1 for patients treated with chemotherapy alone). This represents an important control to more stringently estimate the intrinsic capacity of blasts to form colonies *in vitro*. Unlike LSCs, there was not a significant difference in OS between LPC+ and LPC− patients treated with low-intensity care only (Figure S4C; HR 1.62; 95% CI, 0.79–3.30, p = 0.18). Finally, when we included allogeneic HSCT as a time-dependent variable, non-transplanted LPC+ patients had shorter OS than non-transplanted LPC− patients (solid lines in Figure 3D; HR 2.08; 95% CI, 1.75–2.41); however, there was no significant difference in OS among LPC+ vs. LPC− patients who received HSCT (dotted lines in Figure 3D; HR, 1.28; 95% CI, 0.58–1.98). Similar to the findings in our LSC analysis (Figure 3B), therapeutic effects of HSCT on OS were not significant in either LPC+ or LPC− patients (LPC+ HR 0.76, 95% CI 0.29–1.23; LPC− HR 0.87, 95% CI 0.21–1.53), possibly due to the small number of transplanted patients in each group. Ultimately, high LPC content consistently maintained an association with shorter OS, across subgroup stratification based on therapeutic treatment (Figures 3C and 3D), and when controlling for differences in blast content across patients.

LPCs represented an independent prognostic factor when compared with clinical guidelines of AML patient risk stratification

Prognostic evaluation of patients with AML relies heavily on European Leukemia Net (ELN) guidelines, which stratify patients

(G) Colony counts from serial re-plating experiments outlined in (F). Colony counts are normalized to those of 1° wells. n = 3–17 wells from each of n = 5 patients. Data are shown as mean ± SEM.
$p < 0.05$ is considered statistically significant.
See also Figure S3 and Table S4.

Table 2. Clinical characteristics of 121 patients tested for functional LSC and leukemic progenitor content

	All patients	LPC+	LPC−	LSC+	LSC−
n	121	68	48	52	57
Age					
Years, median (range)	63 (21–94)	63 (24–94)	63 (21–89)	64 (24–89)	59 (21–94)
Gender					
Female (%)	43	40	46	44	42
Blasts					
% median (range)	86.3 (29.8–97.7)	85.7 (29.8–97.7)	87.0 (55–97.2)	89.1 (34–97.7)	82.3 (29.8–97.6)
ELN[a]					
Favorable, n (%)	17 (22)	9 (19)	8 (30)	7 (23)	9 (23)
Intermediate, n (%)	32 (42)	17 (35)	13 (48)	11 (37)	16 (41)
Adverse, n (%)	28 (36)	22 (46)	6 (22)	12 (40)	14 (36)

[a]Information unavailable for one or more patients.

into risk categories based on cytogenetics and mutational status.[26] While others have reported that adverse risk patients are more likely to have readily detectable LSCs at diagnosis,[1] we observed similar performance in both of our functional assays across the three risk categories (Table 2; Figures S4D and S4E). LPCs remained a strong independent prognostic factor in a multivariate Cox proportional hazards model for OS that included ELN 2017 risk stratification, LSC engraftment *in vivo*, and leukemic blast content (Figure 4A). However, LSCs no longer retained their prognostic power when leukemic progenitor content was included in the same model (Figure 4A). During the evolution of our study, updated ELN risk guidelines were released.[27] Based on a manual review of the best available genetic annotation, patients were re-classified based on these updated guidelines. Multivariate analysis with this ELN 2022 classification produced similar results as our initial ELN 2017 analysis that more accurately represented the risk assessment applied at the time of patient management (Table S5). In summary, levels of LPCs have comparatively more powerful prognostic utility to predict patient survival outcomes and do not overlap with conventional ELN risk standards.

Univariate and multivariate Cox modeling were next used to evaluate event-free survival (EFS), which is an important metric because unlike OS, which captures mortality events regardless of whether they are attributable to disease or not, EFS is specific to disease progression/persistence. In previous studies examining relationships between functional LSCs and patient survival outcomes, differences in EFS were either not quite statistically significant between LSC+ vs. LSC− cases[1] or EFS was not reported.[3,4,25] Here, we focused on the patient subgroup that was treated with induction chemotherapy exclusively. Both LSCs and LPCs had a significant negative impact on EFS in univariate models (Figures 4B and 4C; LSC HR 1.82; 95% CI, 1.01–3.27; LPC HR 3.71; 95% CI, 1.98–6.95), including when LPC frequencies were adjusted to account for leukemic blast content (Figure S4F; HR 2.18; 95% CI, 1.22–3.82). However, as seen with OS, multivariate modeling revealed that only leukemic progenitors retained independent prognostic value when combined together with LSCs and ELN risk levels as variables in the model (Figure S4G; Table S6). These analyses represent the most comprehensive assessment of functional progenitors detected by LPCs in relation to AML patient survival and provide a strong demonstration of the prognostic value associated with leukemic progenitor content in adult patients with AML, positioning LPCs as a valuable clinically relevant measure.

DISCUSSION

Based on our results, we propose that cancer stemness is an emergent property[28] that may not reduce to a single molecular network. Recently in the field of AML, clinical measures of stemness have been exemplified by the LSC17 score.[12,13] Our results reinforce the clinically predictive value of this score (Table 1) but also suggest that the genes contributing to the score are preferentially concentrated within $CD34^+$ AML cells. While functional LSCs are often restricted to $CD34^+$ cell fractions, some patients' cells lack CD34 expression entirely (e.g., NPM1+ patients), and their LSCs may be biologically unique from $CD34^+$ LSCs. For example, Quek et al.[29] demonstrated that $CD34^-$ LSCs have distinct transcriptional profiles from LSCs in the $CD34^+$ compartment, and this LSC profile more closely resembled healthy progenitors with granulocytic and macrophage potential. This is consistent with observations by Vergez et al., who have described monocyte progenitor and granulocyte progenitor phenotypes for $CD34^-$ AML cases that harbor NPM1 mutations.[19] Collectively, this suggests challenges in broadly applying a single surrogate phenotype or signature to leukemic stemness as a sole biomarker for patients across various stages of disease management and response. While these signatures continue to evolve,[19,30] functional assays that measure cancer cell behavior and cell fate remain essential until predictions are identified that account for multiple different transcriptional stemness modules or axes.

Given the variability between different assays of cancer stemness available in the field, our study offers leukemic progenitor assays as a reliable measure of functional AML stemness that is both practical and clinically relevant. Xenotransplantation has pioneered the functional assessment of hierarchical organization in AML disease and is considered the gold standard to define leukemic stemness properties. Our findings echo

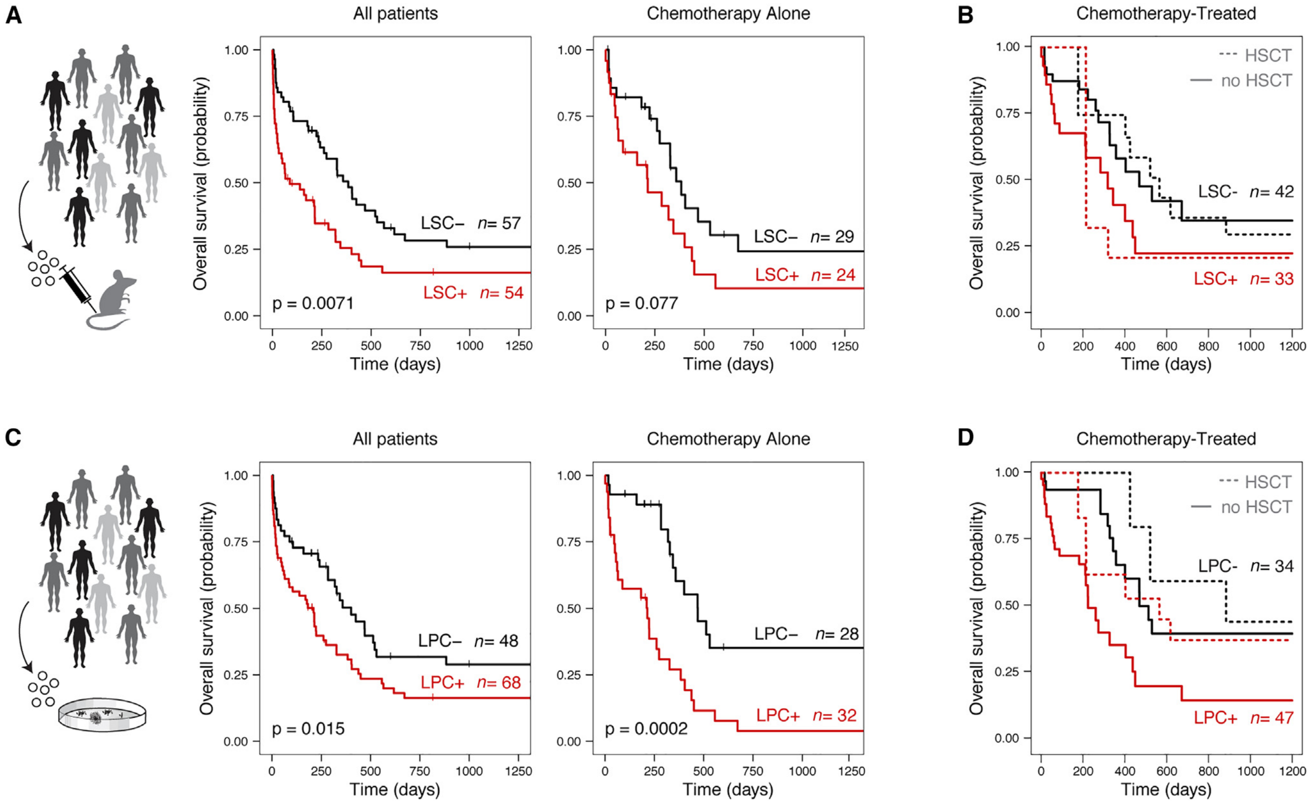

Figure 3. AML patient survival based on functional LSC and LPC content

(A) Kaplan-Meier estimates of overall survival in LSC− and LSC+ patient subsets. Plots display all n = 111 patients tested in xenograft assays (left) or a subset of these patients treated with high-intensity chemotherapy alone (palliative- and HSCT-treated patients excluded; right).

(B) Simon and Makuch estimates of overall survival in LSC− and LSC+ patient subsets, with HSCT as a time-dependent variable (only including patients who were treated with high-intensity chemotherapy, n = 75).

(C) Kaplan-Meier estimates of overall survival in LPC− and LPC+ patient subsets. Plots display all n = 116 patients tested in colony-forming LPC assays (left) or a subset of these patients treated with high-intensity chemotherapy alone (palliative- and HSCT-treated patients excluded; right).

(D) Simon and Makuch estimates of overall survival in LPC− and LPC+ patient subsets, with HSCT as a time-dependent variable (only including patients who were treated with high-intensity chemotherapy, n = 81).

$p < 0.05$ is considered statistically significant.

See also Figure S4 and Tables S5 and S6.

previous observations that human leukemic growth in xenograft assays predicts poor prognosis in patients. Although our results with OS were variable when accounting for differences in therapeutic management across patients, we have established that LSC content has a significant impact on EFS in patients treated with standard induction chemotherapy. In contrast to xenograft-defined LSCs, leukemic progenitors have been previously suggested to correlate with survival[16,31] but, to date, have not been systemically tested against more rigorously defined genetic risk measures. Our results indicate that LPCs provide prognostic value that is not captured by current genetic-based risk assessment[26] or leukemic blast percentages measured at diagnosis.

In the healthy hematopoietic system, clonal tracking studies have suggested that even the healthy hematopoietic system is primarily sustained by the activity of lineage-restricted progenitors with minimal contribution from HSCs,[32] analogous to LPCs and LSCs, respectively. This concept may be applicable to other cancers of the breast, brain, and colon, where CSCs have been detected *in vivo*. The greater sensitivity and reduced cell requirements (several orders of magnitude) for the progenitor assay gives LPC detection an advantage in several applications including drug screening and use in clinical trials of remission induction and relapse follow up, where the sample size attainable from patients is limited. The idea that the measure of progenitors may be equally applicable to other cancers compared with their CSC counterparts detected *in vivo* provides a potential paradigm shift in approaches to define biomarkers or therapeutics in oncology that target cancer stemness.

Limitations of the study

Because the emphasis of our work was to understand patient survival, we prioritized patient samples with the longest possible clinical follow up, which included samples collected as early as 2004. Therefore, a portion of our patient cohort had less extensive genetic characterization at the time of diagnostic

CellPress
OPEN ACCESS

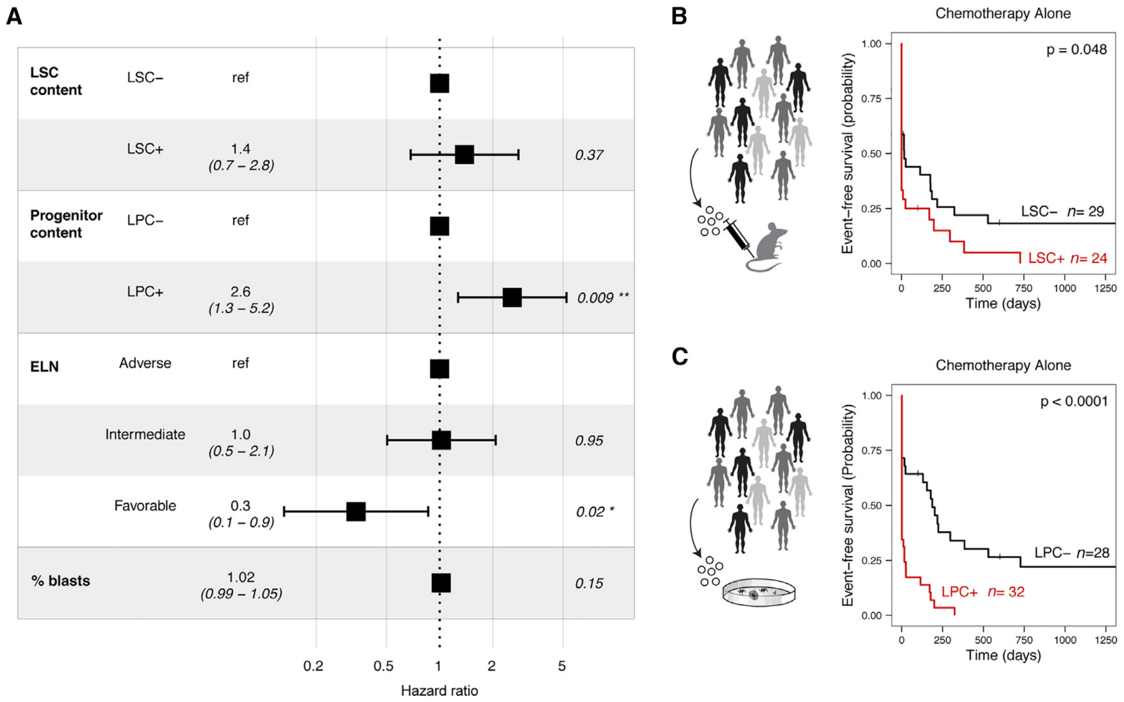

Figure 4. Functional LPC frequencies represent an independent prognostic factor in human AML

(A) Forest plot showing multivariate analysis of overall survival in patients with AML treated with high-intensity chemotherapy alone (n = 60 patients). Unadjusted HRs (squares) and 95% confidence intervals (horizontal lines) are shown.

(B) Kaplan-Meier estimates of event-free survival in LSC− and LSC+ patient subsets (patients treated with high-intensity chemotherapy alone; n = 53).

(C) Kaplan-Meier estimates of event-free survival in LPC− and LPC+ patient subsets (patients treated with high-intensity chemotherapy alone; n = 60).

$p < 0.05$ is considered statistically significant.

See also Figure S4.

assessment, as currently mandated next-generation sequencing panels had not yet been introduced. Retrospective sequencing was performed on cryopreserved cells from some of these earlier samples; however, in some cases, this was not possible due to previous consumption of the sample. It will be important to continue to evaluate our observations in larger cohorts with more exhaustive genetic characterization, particularly as genetic risk assessment tools become more and more refined.[27] In addition, our xenograft assessments were all performed with traditional immune-deficient mouse models that have been shown to produce similar engraftment outcomes.[4] A more recently developed strain of NSG mice has been engineered to express humanized growth factors, and these mice have been shown to be more permissive to human AML engraftment.[33] While it is unclear whether this mouse model is truly reading out the same cell type (i.e., long-term LSCs), it will eventually be interesting to test whether our observations are sustained when using these adapted *in vivo* xenograft models.

STAR★METHODS

Detailed methods are provided in the online version of this paper and include the following:

- KEY RESOURCES TABLE
- RESOURCE AVAILABILITY
  - Lead contact
  - Materials availability
  - Data and code availability
- EXPERIMENTAL MODEL AND SUBJECT DETAILS
  - Primary human hematopoietic samples
  - Murine recipients for xenograft assays
- METHOD DETAILS
  - Xenotransplantation
  - Serial transplantation
  - Single cell RNA sequencing
  - Nanostring assay
  - Methylcellulose progenitor assays
  - Serial re-plating
  - Fluorescence-activated cell sorting (FACS) and flow cytometry
  - Droplet digital polymerase chain reaction
- QUANTIFICATION AND STATISTICAL ANALYSIS

SUPPLEMENTAL INFORMATION

Supplemental information can be found online at https://doi.org/10.1016/j.xcrm.2023.101108.

ACKNOWLEDGMENTS

The authors would like to thank Marie-Pierre Mathieu, Aline Comyn, and Emily Broder for their assistance with xenotransplantation experiments. They would

also like to acknowledge Tracey Murphy and colleagues at the Princess Margaret Cancer Center for running LSC17 NanoString assays, Maria Ines Pinto Sanchez for guidance and preliminary statistical analysis, and Tara Paton and Guillermo Casallo for assistance with ddPCR. This work was supported by the Canadian Cancer Society (grant 703204) and Canadian Institutes of Health Research (CIHR-FRN #159925), as well as the David Braley Stem Cell Library. M.B. is supported by the Canadian Chair Program and holds the Canada Research Chair in human stem cell biology and the Michael DeGroote Chair in Human Stem Cell Biology and Cancer Research.

AUTHOR CONTRIBUTIONS

A.L.B. performed experiments, analyzed single-cell transcriptomics and survival data, and wrote the manuscript; J.L. performed experiments and performed analysis; W.Y. performed experiments and reviewed clinical data; C.G.H., J.C.R., L.A., S.M., S.V., P.J., A.E., D.P.P., and A.L. performed experiments; S.M. performed analysis; D.X. and E.K. reviewed clinical data; R.F. and M.T. provided patient samples; B.L. and A.X. provided patient samples and provided input on the manuscript; M.B. designed the study and wrote the manuscript.

DECLARATION OF INTERESTS

The authors declare no competing interests.

Received: August 12, 2022
Revised: January 29, 2023
Accepted: June 16, 2023
Published: July 10, 2023

REFERENCES

1. Pearce, D.J., Taussig, D., Zibara, K., Smith, L.L., Ridler, C.M., Preudhomme, C., Young, B.D., Rohatiner, A.Z., Lister, T.A., and Bonnet, D. (2006). AML engraftment in the NOD/SCID assay reflects the outcome of AML: implications for our understanding of the heterogeneity of AML. Blood *107*, 1166–1173.
2. Shlush, L.I., Mitchell, A., Heisler, L., Abelson, S., Ng, S.W.K., Trotman-Grant, A., Medeiros, J.J.F., Rao-Bhatia, A., Jaciw-Zurakowsky, I., Marke, R., et al. (2017). Tracing the origins of relapse in acute myeloid leukaemia to stem cells. Nature *547*, 104–108.
3. Pabst, C., Bergeron, A., Lavallée, V.P., Yeh, J., Gendron, P., Norddahl, G.L., Krosl, J., Boivin, I., Deneault, E., Simard, J., et al. (2016). GPR56 identifies primary human acute myeloid leukemia cells with high repopulating potential in vivo. Blood *127*, 2018–2027.
4. Vargaftig, J., Taussig, D.C., Griessinger, E., Anjos-Afonso, F., Lister, T.A., Cavenagh, J., Oakervee, H., Gribben, J., and Bonnet, D. (2012). Frequency of leukemic initiating cells does not depend on the xenotransplantation model used. Leukemia *26*, 858–860.
5. Bonnet, D., and Dick, J.E. (1997). Human acute myeloid leukemia is organized as a hierarchy that originates from a primitive hematopoietic cell. Nat. Med. *3*, 730–737.
6. Lapidot, T., Sirard, C., Vormoor, J., Murdoch, B., Hoang, T., Caceres-Cortes, J., Minden, M., Paterson, B., Caligiuri, M.A., and Dick, J.E. (1994). A cell initiating human acute myeloid leukaemia after transplantation into SCID mice. Nature *367*, 645–648.
7. Terwijn, M., Zeijlemaker, W., Kelder, A., Rutten, A.P., Snel, A.N., Scholten, W.J., Pabst, T., Verhoef, G., Löwenberg, B., Zweegman, S., et al. (2014). Leukemic stem cell frequency: a strong biomarker for clinical outcome in acute myeloid leukemia. PLoS One *9*, e107587.
8. van Rhenen, A., Feller, N., Kelder, A., Westra, A.H., Rombouts, E., Zweegman, S., van der Pol, M.A., Waisfisz, Q., Ossenkoppele, G.J., and Schuurhuis, G.J. (2005). High stem cell frequency in acute myeloid leukemia at diagnosis predicts high minimal residual disease and poor survival. Clin. Cancer Res. *11*, 6520–6527.
9. Eppert, K., Takenaka, K., Lechman, E.R., Waldron, L., Nilsson, B., van Galen, P., Metzeler, K.H., Poeppl, A., Ling, V., Beyene, J., et al. (2011). Stem cell gene expression programs influence clinical outcome in human leukemia. Nat. Med. *17*, 1086–1093.
10. Sarry, J.E., Murphy, K., Perry, R., Sanchez, P.V., Secreto, A., Keefer, C., Swider, C.R., Strzelecki, A.C., Cavelier, C., Récher, C., et al. (2011). Human acute myelogenous leukemia stem cells are rare and heterogeneous when assayed in NOD/SCID/IL2Rgammac-deficient mice. J. Clin. Invest. *121*, 384–395.
11. Taussig, D.C., Vargaftig, J., Miraki-Moud, F., Griessinger, E., Sharrock, K., Luke, T., Lillington, D., Oakervee, H., Cavenagh, J., Agrawal, S.G., et al. (2010). Leukemia-initiating cells from some acute myeloid leukemia patients with mutated nucleophosmin reside in the CD34(-) fraction. Blood *115*, 1976–1984.
12. Ng, S.W.K., Murphy, T., King, I., Zhang, T., Mah, M., Lu, Z., Stickle, N., Ibrahimova, N., Arruda, A., Mitchell, A., et al. (2022). A clinical laboratory-developed LSC17 stemness score assay for rapid risk assessment of patients with acute myeloid leukemia. Blood Adv. *6*, 1064–1073.
13. Ng, S.W.K., Mitchell, A., Kennedy, J.A., Chen, W.C., McLeod, J., Ibrahimova, N., Arruda, A., Popescu, A., Gupta, V., Schimmer, A.D., et al. (2016). A 17-gene stemness score for rapid determination of risk in acute leukaemia. Nature *540*, 433–437.
14. Blair, A., Hogge, D.E., and Sutherland, H.J. (1998). Most acute myeloid leukemia progenitor cells with long-term proliferative ability in vitro and in vivo have the phenotype CD34(+)/CD71(-)/HLA-DR. Blood *92*, 4325–4335.
15. Sutherland, H.J., Blair, A., and Zapf, R.W. (1996). Characterization of a hierarchy in human acute myeloid leukemia progenitor cells. Blood *87*, 4754–4761.
16. Delmer, A., Marie, J.P., Thevenin, D., Cadiou, M., Viguié, F., and Zittoun, R. (1989). Multivariate analysis of prognostic factors in acute myeloid leukemia: value of clonogenic leukemic cell properties. J. Clin. Oncol. *7*, 738–746.
17. Curtis, J.E., Messner, H.A., Hasselback, R., Elhakim, T.M., and McCulloch, E.A. (1984). Contributions of host- and disease-related attributes to the outcome of patients with acute myelogenous leukemia. J. Clin. Oncol. *2*, 253–259.
18. McCulloch, E.A., Curtis, J.E., Messner, H.A., Senn, J.S., and Germanson, T.P. (1982). The contribution of blast cell properties to outcome variation in acute myeloblastic leukemia (AML). Blood *59*, 601–608.
19. Vergez, F., Largeaud, L., Bertoli, S., Nicolau, M.L., Rieu, J.B., Vergnolle, I., Saland, E., Sarry, A., Tavitian, S., Huguet, F., et al. (2022). Phenotypically-defined stages of leukemia arrest predict main driver mutations subgroups, and outcome in acute myeloid leukemia. Blood Cancer J. *12*, 117–20220816.
20. Jaatinen, T., Hemmoranta, H., Hautaniemi, S., Niemi, J., Nicorici, D., Laine, J., Yli-Harja, O., and Partanen, J. (2006). Global gene expression profile of human cord blood-derived CD133+ cells. Stem Cell. *24*, 631–641.
21. Gal, H., Amariglio, N., Trakhtenbrot, L., Jacob-Hirsh, J., Margalit, O., Avigdor, A., Nagler, A., Tavor, S., Ein-Dor, L., Lapidot, T., et al. (2006). Gene expression profiles of AML derived stem cells; similarity to hematopoietic stem cells. Leukemia *20*, 2147–2154.
22. Aslostovar, L., Boyd, A.L., Benoit, Y.D., Di Lu, J., Garcia Rodriguez, J.L., Nakanishi, M., Porras, D.P., Reid, J.C., Mitchell, R.R., Leber, B., et al. (2021). Abnormal dopamine receptor signaling allows selective therapeutic targeting of neoplastic progenitors in AML patients. Cell Rep. Med. *2*, 100202–20210216.
23. Shlush, L.I., Zandi, S., Mitchell, A., Chen, W.C., Brandwein, J.M., Gupta, V., Kennedy, J.A., Schimmer, A.D., Schuh, A.C., Yee, K.W., et al. (2014). Identification of pre-leukaemic haematopoietic stem cells in acute leukaemia. Nature *506*, 328–333.

CellPress
OPEN ACCESS

24. Lee, J.H., Salci, K.R., Reid, J.C., Orlando, L., Tanasijevic, B., Shapovalova, Z., and Bhatia, M. (2017). Brief Report: Human Acute Myeloid Leukemia Reprogramming to Pluripotency Is a Rare Event and Selects for Patient Hematopoietic Cells Devoid of Leukemic Mutations. Stem Cell. *35*, 2095–2102.

25. Griessinger, E., Vargaftig, J., Horswell, S., Taussig, D.C., Gribben, J., and Bonnet, D. (2018). Acute myeloid leukemia xenograft success prediction: Saving time. Exp. Hematol. *59*, 66–71.e4.

26. Döhner, H., Estey, E., Grimwade, D., Amadori, S., Appelbaum, F.R., Büchner, T., Dombret, H., Ebert, B.L., Fenaux, P., Larson, R.A., et al. (2017). Diagnosis and management of AML in adults: 2017 ELN recommendations from an international expert panel. Blood *129*, 424–447.

27. Dohner, H., Wei, A.H., Appelbaum, F.R., Craddock, C., DiNardo, C.D., Dombret, H., Ebert, B.L., Fenaux, P., Godley, L.A., Hasserjian, R.P., et al. (2022). Diagnosis and Management of AML in Adults: 2022 ELN Recommendations from an International Expert Panel. Blood.

28. Prager, B.C., Xie, Q., Bao, S., and Rich, J.N. (2019). Cancer Stem Cells: The Architects of the Tumor Ecosystem. Cell Stem Cell *24*, 41–53.

29. Quek, L., Otto, G.W., Garnett, C., Lhermitte, L., Karamitros, D., Stoilova, B., Lau, I.J., Doondeea, J., Usukhbayar, B., Kennedy, A., et al. (2016). Genetically distinct leukemic stem cells in human CD34- acute myeloid leukemia are arrested at a hemopoietic precursor-like stage. J. Exp. Med. *213*, 1513–1535.

30. Zeng, A.G.X., Bansal, S., Jin, L., Mitchell, A., Chen, W.C., Abbas, H.A., Chan-Seng-Yue, M., Voisin, V., van Galen, P., Tierens, A., et al. (2022). A cellular hierarchy framework for understanding heterogeneity and predicting drug response in acute myeloid leukemia. Nat. Med. *28*, 1212–1223.

31. Sutherland, H., Blair, A., Vercauteren, S., and Zapf, R. (2001). Detection and clinical significance of human acute myeloid leukaemia progenitors capable of long-term proliferation in vitro. Br. J. Haematol. *114*, 296–306.

32. Sun, J., Ramos, A., Chapman, B., Johnnidis, J.B., Le, L., Ho, Y.J., Klein, A., Hofmann, O., and Camargo, F.D. (2014). Clonal dynamics of native haematopoiesis. Nature *514*, 322–327.

33. Krevvata, M., Shan, X., Zhou, C., Dos Santos, C., Habineza Ndikuyeze, G., Secreto, A., Glover, J., Trotman, W., Brake-Silla, G., Nunez-Cruz, S., et al. (2018). Cytokines increase engraftment of human acute myeloid leukemia cells in immunocompromised mice but not engraftment of human myelodysplastic syndrome cells. Haematologica *103*, 959–971.

34. Stuart, T., Butler, A., Hoffman, P., Hafemeister, C., Papalexi, E., Mauck, W.M., 3rd, Hao, Y., Stoeckius, M., Smibert, P., and Satija, R. (2019). Comprehensive Integration of Single-Cell Data. Cell *177*, 1888–1902.e21.

35. Lun, A.T.L., Bach, K., and Marioni, J.C. (2016). Pooling across cells to normalize single-cell RNA sequencing data with many zero counts. Genome Biol. *17*, 75.

36. Therneau, T.M., and Grambsch, P.M. (2000). Modeling Survival Data: Extending the Cox Model, 1st edition (Springer).

37. van Buuren, S., and Groothuis-Oudshoorn, K. (2011). mice: Multivariate Imputation by Chained Equations in R. J. Stat. Software *45*, 1–67.

38. Hu, Y., and Smyth, G.K. (2009). ELDA: extreme limiting dilution analysis for comparing depleted and enriched populations in stem cell and other assays. J. Immunol. Methods *347*, 70–78.

39. Boyd, A.L., Aslostovar, L., Reid, J., Ye, W., Tanasijevic, B., Porras, D.P., Shapovalova, Z., Almakadi, M., Foley, R., Leber, B., et al. (2018). Identification of Chemotherapy-Induced Leukemic-Regenerating Cells Reveals a Transient Vulnerability of Human AML Recurrence. Cancer Cell *34*, 483–498.e5.

40. Döhner, K., Thiede, C., Jahn, N., Panina, E., Gambietz, A., Larson, R.A., Prior, T.W., Marcucci, G., Jones, D., Krauter, J., et al. (2020). Impact of NPM1/FLT3-ITD genotypes defined by the 2017 European LeukemiaNet in patients with acute myeloid leukemia. Blood *135*, 371–380.

STAR★METHODS

KEY RESOURCES TABLE

REAGENT or RESOURCE	SOURCE	IDENTIFIER
**Antibodies**		
APC mouse anti-human CD34	BD Pharmingen	Cat#555824; RRID:AB_398614
V450 mouse anti-human CD45	BD Horizon	Cat#642275; RRID:AB_1645755
APC mouse anti-human CD33	BD Pharmingen	Cat#551378; RRID:AB_398502
FITC mouse anti-human CD19	BD Pharmingen	Cat#555412; RRID:AB_395812
**Biological samples**		
Primary AML patient samples	Juravinksi hospital and Cancer Center London Health Sciences Center	N/A
Healthy human donor hematopoietic samples	McMaster Boris Clinic	N/A
**Chemicals, peptides, and recombinant proteins**		
7AAD	Beckman Coulter	Item#A07704
**Critical commercial assays**		
Total RNA purification kit	Norgen Biotek	Cat#37500
DNeasy blood and tissue kit	Qiagen	Cat#69506
QIAamp DNA micro kit	Qiagen	Cat#56304
Methocult	Stemcell technologies	Cat#H4434
T cell Positive Selection Kit	Stemcell Technologies	Cat#17851
Chromium Single Cell 3′ Library & Gel Bead Kit v2	10X Genomics	Cat#120267
Chromium Single Cell A Chip Kit	10X Genomics	Cat#1000009
i7 Multiplex Kit	10X Genomics	Cat#120262
ddPCR^{TM} Supermix for Probes	BioRad Laboratories	Cat#1863010
**Experimental models: Organisms/strains**		
NOD.CB17-*Prkdc*^{scid}/J	The Jackson laboratory	RRID:IMSR_JAX:001303
NOD*scid Il2ry*^{null}*B2m*^{null}	The Jackson laboratory	RRIS:IMSR JAX:010636
NOD.Cg-*Prkdc*^{scid}Il2rg^{tm1wjl}/SzJ	The Jackson laboratory	RRID:IMSR_ARC:NSG JAX:05557
**Deposited data**		
Raw scRNAseq data	This study	GSE234743
**Software and algorithms**		
FACSDiva	BD	http://www.bdbiosciences.com/us/instruments/research/software/flow-cytometry-acquisition
FlowJo10	FlowJo, LLC	https://www.flowjo.com
CellRanger	10X Genomics v3.1.0	https://support.10xgenomics.com/single-cell-gene-expression/software/overview/welcome
Prism v5.0a	Graphpad	https://www.graphpad.com/scientific-software/prism/
Columbus	Perkin Elmer	https://www.perkinelmer.com/product/image-data-storage-and-analysis-system-columbus
Acapella	Perkin Elmer	N/A
MedCalc v20.110	MedCalc Software Ltd	https://www.medcalc.org/calc/diagnostic_test.php
QuantaSoft Analysis Pro v1.0.596	BioRad Laboratories	www.bio-rad.com
R v3.5.1	R Core	https://www.r-project.com
Seurat v3.1.1	Stuart et al.[34]	https://github.com/satijalab/seurat/releases/tag/v3.1.1

(*Continued on next page*)

Continued

REAGENT or RESOURCE	SOURCE	IDENTIFIER
scran v1.14.6	Lun et al.[35]	https://bioconductor.org/packages/release/bioc/html/scran.html
survival v3.2-7	Therneau[36]	https://CRAN.R-project.org/package=survival.
survminer v0.4.8	N/A	https://github.com/kassambara/survminer
mice v3.11.0	Van Buuren[37]	https://cran.r-project.org/package=mice

RESOURCE AVAILABILITY

Lead contact

Further information and requests for resources and reagents should be directed to and will be fulfilled by the corresponding author, Mickie Bhatia (mbhatia@mcmaster.ca).

Materials availability

This study did not generate new unique reagents.

Data and code availability

- Single-cell RNA-seq data have been deposited at GEO and are publicly available as of the date of publication. Accession numbers are listed in the key resources table.
- This paper does not report original code.
- Any information or resources required to reanalyze the data reported in this paper is available from the lead contact upon request.

EXPERIMENTAL MODEL AND SUBJECT DETAILS

Primary human hematopoietic samples

121 non-APL AML patients were enrolled in our study at two separate clinical sites (Juravinski Hospital and Cancer Center in Hamilton, Ontario and London Health Sciences Center in London, Ontario). Informed consent was obtained from all sample donors in accordance with Research Ethics Board-approved protocols at McMaster University (Hamilton Integrated Research Ethics Board) and the London Health Sciences Center (Western University Research Ethics Board). Details of AML patients are outlined in Table 2. The majority of patients who were treated with curative intent received a 3 + 7 induction chemotherapy protocol (intravenous cytarabine 200 mg/m^2/day for 7 days with daunorubicin 60 mg/m^2 for days 1–3). Nine patients were treated with a regimen of cytarabine and mitoxantrone (mitoxantrone 12 mg/m^2/day for 5 days with cytarabine 1000 mg/m^2 q12h on days 1–3). Primary AML specimens were obtained from peripheral blood apheresis or BM aspirates.

Normal peripheral blood samples were also collected from healthy donors at McMaster University. All samples were obtained from consenting donors in accordance with approved protocols by the Research Ethics Board at McMaster University and the London Health Sciences Center, University of Western Ontario. Mononuclear cells were recovered by density gradient centrifugation (Ficoll-Paque Premium; GE Healthcare) followed by red blood cell lysis using ammonium chloride solution (Stemcell Technologies).

Murine recipients for xenograft assays

Mice were bred and maintained at the McMaster Animal Barrier Facility. Animals were housed in groups of 2–5 mice per cage. All experimental procedures were approved by the Animal Council of McMaster University. Immune deficient mice were used as human-mouse xenograft recipients, all based on the NOD/SCID parental line (NOD. Cg-Prkdc^{scid}). A total of 3 substrains were used to fully analyze LSC potential from AML patient samples, including with or without IL2 receptor common gamma chain (*IL2rg*^{null}; i.e., NOD/SCID and NSG strains) or additional deletion of MHC I beta-2 microglobulin (*B2m*^{null}). Previous studies have established that engraftment outcomes are consistent between these mouse strains.[4]

METHOD DETAILS

Xenotransplantation

Across strains, 6–10 week old mice were sublethally irradiated (315–350 Rads, using a 137Cs γ-irradiator), 24 h prior to transplantation of primary AML patient samples via intra-venous or intra-femoral injection.[5,6] CD3$^+$ cell depletion was performed prior to transplantation into *IL2rg*^{null} NOD/SCID mice (NSGs), using a commercially available T cell magnetic selection kit (Miltenyi Biosciences). Our standard procedure was to transplant a minimum of 3 mice at 5 million cells per recipient, for a total of 368 mice analyzed in total. Occasionally, mice suffered from radiation sickness requiring early euthanasia, and these were excluded from human chimerism

analysis. At least 4 weeks following transplantation, BM cells of recipient mice were recovered by mechanical dissociation and analyzed by flow cytometry. Criteria for leukemic engraftment was ≥0.1% human $CD45^+$ with a composition ≥75% $CD33^+$ within the human graft. Mice with evidence of healthy human hematopoeitic reconstitution ($CD19^+$ cells ≥25% of human graft) were considered negative for LSC content.

Serial transplantation

To demonstrate the property of self-renewal, BM from 1° xenograft recipients was serially transplanted into 2° recipient mice of the same strain, by IV transplantation (Table S3). In most cases, the entire collection of BM cells recovered from an individual 1° recipient mouse was transplanted into a single 2° recipient (''mouse-to-mouse''). In other cases, BM cells were pooled across 1° recipient mice and transplanted into multiple 2° recipients (''pooled''). Positive leukemic engraftment was seen in 2° recipients for all 9/9 patients tested. In two cases, we quantified functional LSC content in 1° NSG xenograft recipients by limiting dilution transplantation into 2° NSG recipient mice. Human AML cells were injected IV into secondary mice at a range of different cell doses (10k to 300k cells for AML#2, 260k to 2.6×10^6 for AML#8). For AML#2, 4 secondary mice were transplanted for each of 5 cell doses, and for AML#8, up to 3 secondary mice were transplanted for each of 3 cell doses. Functional LSC frequencies were estimated using ELDA software.[38]

Single cell RNA sequencing

Xenografted scRNAseq datasets were derived from grafts that produced exclusively myeloid human cells, and these cells were FACS-purified based on human $CD45^+CD33^+$ expression. For *de novo* patient samples analyzed, next generation sequencing data was available for 3/4 patients, which confirmed that pathogenic driver mutations were found at variant allele frequencies ≥47% for all 3 patients (TP53, NPM1, or ETV6). This assured us that each sample was primarily comprised of mutant leukemic cells. We lacked genetic characterization for 1/4 patient samples analyzed. For this sample, we performed leukemic blast purification by FACS prior to performing scRNAseq. For all samples, 7AAD exclusion was used to remove dead cells and debris prior to scRNAseq. Single cells were then captured using a Chromium Controller system (10X Genomics) and barcoded cDNA libraries were prepared using the Chromium Single Cell 3′ Library and Gel Bead kit (10X Genomics, v2 or v3), following the manufacturer's instructions. Indexed libraries were sequenced on a HiSeq2500 or NovaSeq platform (Illumina, 100 bp paired-end) and raw Illumina files were demultiplexed and aligned to GRCh38 reference using the CellRanger pipeline (*mkfastq* and *count*, 10x GENOMICS, v3.1.0 or v6.0.2). Across samples, this yielded ≥20K reads per cell; ≥1,400 median genes per cell; and ≥3.5K median UMI counts per cell. Subsequent quality control and normalization steps were performed in R (3.6.0) using the packages *scater* and *scran* (v1.14.6). Briefly, doublets and poor-quality cells were removed if they were outliers based on gene number, UMI number, or the percentage of mitochondrial genes (median absolute deviation >3). Our final processed datasets included >2500 cells each. For data visualization, we used R packages *Seurat* (v3.1.1) and *ggridges* (v0.5.3). All 17 genes from the LSC17 score were detected across datasets, with the exception of DPYSL3 for Patient #2 xenograft cells and ADGRG1 for Patient #20 *de novo* cells. Using scaled and log-normalized transcript expression values, single cell LSC17 scores were calculated using the following formula[12]: 0.00582*LAPTM4B - 0.0347*ZBTB46–0.0138*ARHGAP22 + 0.0465*BEX3 + 0.0874*DNMT3B + 0.0258*MMRN1 + 0.0271*SOCS2 - 0.0402*AKR1C3 -0.0704*CDK6 - 0.0258*CPXM1 + 0.00865*NYNRIN +0.0146*EMP1 + 0.0196*FAM30A + 0.0338*CD34 + 0.0501*ADGRG1 + 0.0284*DPYSL3 - 0.0226*SMIM24. Scores for published LSC, HSC, and progenitor signatures[9,20,21] were calculated using Seurat's *AddModuleScore* function. Uniform Manifold Approximation and Projection (UMAP) was used for dimensionality reduction and to visualize CD34 and LSC17 score distributions of individual samples. In order to visualize cellular heterogeneity of samples, all six datasets were merged together followed by re-scaling with cell cycle regression. Clustering was performed for the combined dataset as a whole, as well as linear dimensionality reduction using Principal Component Analysis.

Nanostring assay

RNA was isolated from human cell populations using a total RNA purification kit (Norgen Biotek) according to the manufacturer's instructions. A custom Nanostring nCounter Assay was applied as previously described.[12] LSC17 scores ranged from −0.18 to 1.18, and were classified as high or low based on comparison to median LSC17 scores of a reference cohort described in Ng et al.[12]

Methylcellulose progenitor assays

The frequency of leukemic progenitors (LPCs) was evaluated by colony-forming unit (CFU) assays. Briefly, AML cells were seeded in semisolid methylcellulose media (Methocult GF #H4434; Stemcell Technologies) at densities of 1,000–50,000 cells/well according to established protocols.[39] Following 7–14 days, colonies were manually scored and/or fluorescent images were captured for automated colony quantification. CFU wells were stained with calcein green fluorescent dye and whole well images were acquired at 2x using the Operetta High Content Screening platform (Perkin Elmer) by means of epi-fluorescence illumination and standard filter sets. Stitched whole well images were constructed in Columbus software (Perkin Elmer). Custom image analysis scripts were used to identify and quantify individual colonies (Acapella software; Perkin Elmer). We required a minimum of 15 cells to score leukemic colonies, similar to criteria applied by Vergez et al.[19] LPC#s per 10,000 cells were calculated and AML patient samples were stratified into LPC+ vs. LPC- groups based on a threshold of >3 colonies/10k or ≤3 colonies/10k, as healthy donor mononuclear cells produced an average of 3 colonies per 10k cells (n = 3). While plating of bulk samples is the most practical scalable method to quantify

CellPress
OPEN ACCESS

LPCs, we have also calculated the LPC#s per 10,000 leukemic blasts to estimate the intrinsic capacity of diseased blast cells to form colonies *in vitro*.

Serial re-plating

Serial re-plating experiments were performed for 5 AML samples to evaluate the ability of LPCs to self-renew. Following colony formation over a 14-day period, colonies were manually counted, and then entire well contents were collected, and colonies were dissociated into single cell suspensions. Single cell suspensions from whole 1° CFU wells were then each plated into individual 2° CFU wells following the same established methods, and 2° colonies were counted after 14 days.

Fluorescence-activated cell sorting (FACS) and flow cytometry

Immunophenotyping for human hematopoietic cell surface markers was carried out using the following antibodies: V450-conjugated anti-CD45 (1:100; 2D1), APC-conjugated anti-CD33 (1:300; WM-33), APC-conjugated anti-CD34 (1:200; 581), or FITC-conjugated anti-CD19 (1:100; HIB19; all from BD). FACS sorting was performed using a FACSAria II sorter, and flow cytometry analysis was performed with an LSRII Cytometer (BD). FACSDiva (BD) and software was used for data acquisition and FlowJo software (Tree Star) was used for analysis. For survival analysis, AML patients were stratified into CD34high vs. CD34low groups based on a threshold of 50% $CD34^+$ cells.

Droplet digital polymerase chain reaction

DNA extractions were performed using Qiagen DNeasy kits (AML mononuclear cells) or QIAamp DNA micro kits (colonies individually plucked from CFU assays), according to the manufacturer's instructions. ddPCR was performed on the QX200 Droplet Digital PCR system (Bio-Rad Laboratories, Inc., Hercules, CA, USA) using custom (Table S4) or commercial (NPM1, Hs000000064_rm) or custom TaqMan SNP Genotyping assays (Life Technologies, Carlsbad, CA, USA). The 20 μl reaction mix consisted of 10 μL of 2x ddPCR SuperMix for Probes (Bio-Rad Laboratories), 0.5 μL of the 40X assay, 6.5–9.5 μL water and 1–4 μL of 20-50 ng/μl genomic DNA. The assay was tested by temperature gradient to ensure optimal separation of reference and variant signals. Cycling conditions for the reactions were 95C for 10 min, followed by 45 cycles of 94C for 30 s and 60C for 1 min, 98C for 10 min and finally a 4C hold on a Life Technologies Veriti thermal cycler. Data was analyzed using QuantaSoft Analysis Pro software v1.0.596 (Bio-Rad Laboratories).

QUANTIFICATION AND STATISTICAL ANALYSIS

MedCalc software (v20.110) was used to calculate sensitivity, specificity, predictive values, and likelihood ratios. All other analyses were performed in Prism software (v5.0a; GraphPad) or R v3.5.1 (using packages *survival* v3.2-7, *survminer* v0.4.8, *mice* v3.11.0, and *stats* 3.5.1). Survival times were calculated from the date of sample collection, using previously established criteria for OS and EFS[1]; by subtracting the date of sample collection from the date of death (OS) or from the date of relapse (EFS). Patients with primary refractory disease were assigned an EFS of 0 days. The Kaplan Meier method was used for univariate survival analyses and multivariate Cox regression was used to evaluate independent predictors of survival. We also evaluated allogeneic HSCT as a time-dependent variable in univariate Cox models.[40] For multivariate survival analyses, missing data were replaced by Bayesian polytomous regression or logistic regression. Pearson's product moment correlation was used for correlation analyses and paired or unpaired Student's t-tests were used to compare quantitative data between groups.

Cell Reports Medicine, Volume *4*

Supplemental information

Leukemic progenitor compartment serves

as a prognostic measure of cancer stemness

in patients with acute myeloid leukemia

Allison L. Boyd, Justin Lu, Cameron G. Hollands, Lili Alsostovar, Shiva Murali, Jennifer C. Reid, Wendy Ye, Sean Vandersluis, Paige Johnson, Amro ElRafie, Deanna P. Porras, Dimetri Xenocostas, Andrew Leber, Brian Leber, Ronan Foley, Michael Trus, Tobias Berg, Eri Kawata, Anargyros Xenocostas, and Mickie Bhatia

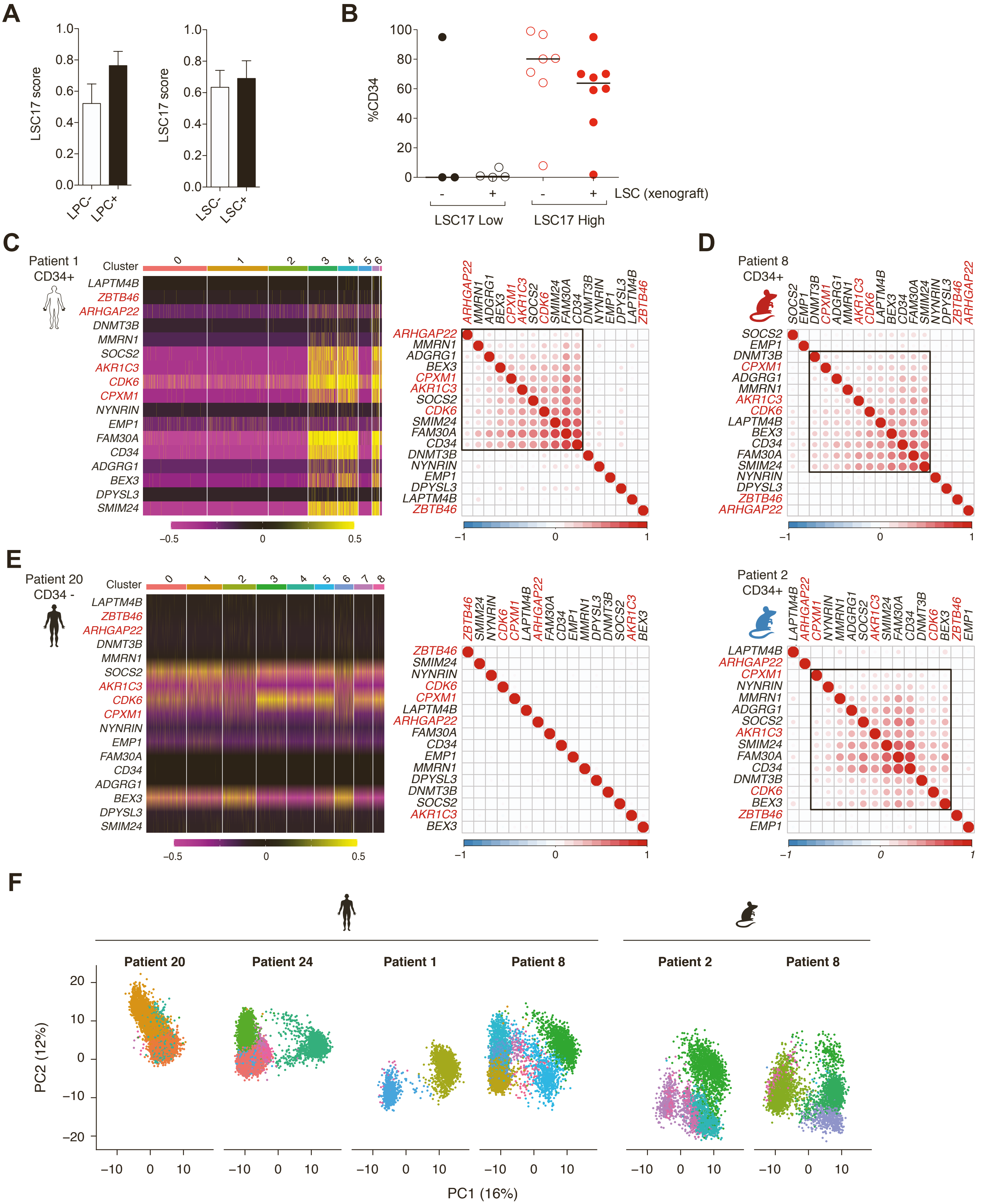

Figure S1. LSC17 scores and single cell LSC17 gene expression in human AML. Related to Figure 1.
(A) Comparison of LSC17 transcriptional scores in LPC- vs. LPC+ or LSC- vs. LSC+ patient subsets (n=22 patients total). LSC17 scores were derived from bulk RNA analyzed using the Nanostring platform. (B) Percentage CD34 expression measured by flow cytometry (n=22 patients). Black coloring indicates LSC17^{Low} and red coloring indicates LS17^{High}. Solid data points indicate similar classification between LSC17 scores and xenograft assays while hollow data points indicate different classification between LSC17 scores and xenograft assays.

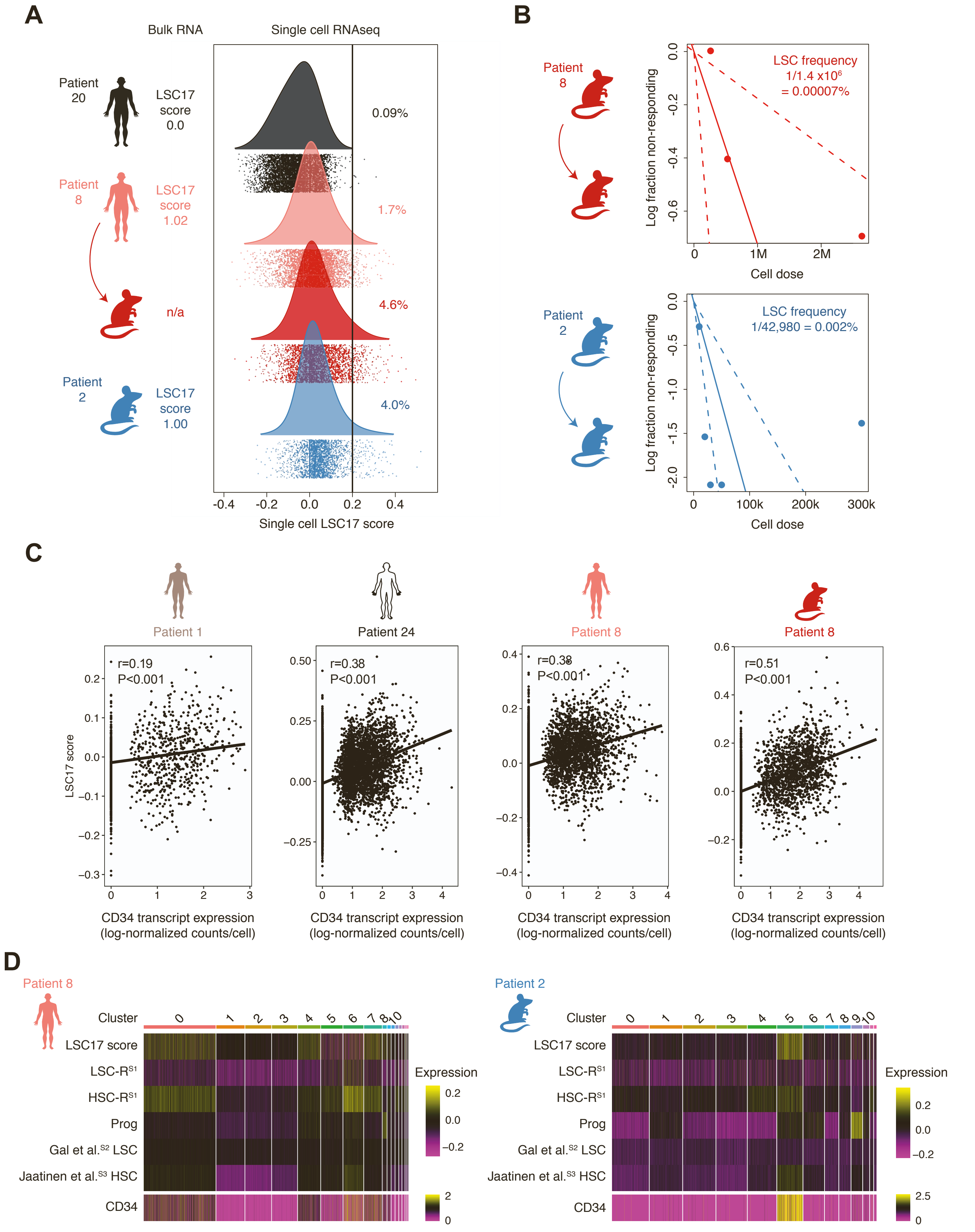

Figure S2. LSC17 scores correlate with CD34 expression at the single cell level. Related to Figure 1.
(A) Histogram showing the distribution of LSC17 transcriptional scores across individual human cells from AML Patient #20 and AML Patient #8, as well as xenografts from AML Patient #8 and AML Patient #2 (FACS-purified for human CD45+CD33+ cells). LSC17 scores derived from bulk RNA are shown for 3 of the samples. Single cell LSC17 score threshold of 0.2 was defined based on the histogram for Patient #20 (LSC17 score = 0 in bulk RNA assay for this patient).

(B) Limiting dilution serial transplantation to quantify functional LSC frequencies in xenografted human cells from AML Patient #2 and AML Patient #8. Both experiments involved IV transplantation of BM cells from 1° NSG recipients into 2° NSG recipients. (C) Correlation between CD34 transcript expression and LSC17 scores in single human AML cells. (D) Heat maps of LSC17 scores and published signatures of LSC/HSC/progenitors in single cells from an AML patient (Patient #8) and purified human cells from an AML-xenograft (Patient #2). LSC-R, HSC-R and Prog (progenitor) signatures are from Eppert et al.[S1], and additional LSC and HSC signatures were obtained from Gal et al.[S2] and Jaatinen et al.[S3], respectively.

A

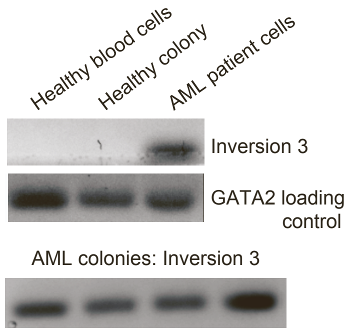

C

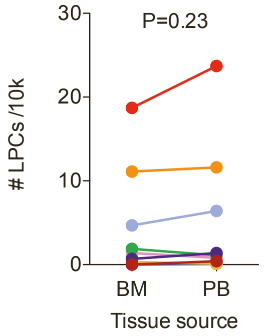

D

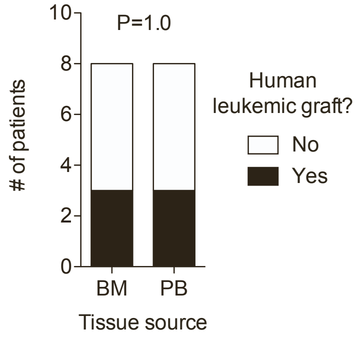

B

Source	# Colonies	Mutation	Detection technique	# Mutant colonies	# Wildtype colonies
Aslostovar et al[S4]	10	-7	ddPCR	10	0
Aslostovar et al[S4]	8	FLT3-ITD	PCR	7	1
Lee et al[S5]	1	MLL-AF9	FISH	25 cells from pooled colonies	0 cells
Patient 20	4	DNMT3A<br>ETV6	ddPCR<br>ddPCR	4<br>3	0<br>1
Patient 105	5	DNMT3A<br>NPM1	ddPCR<br>ddPCR	5<br>5	0<br>0
Patient 8	10	TP53<br>GRM8	ddPCR<br>ddPCR	10<br>10	0<br>0
Patient 106	5	TET2<br>NRAS	ddPCR<br>ddPCR	5<br>5	0<br>0
Patient 107	3	IDH2	ddPCR	3	0
Patient 108	3	ZRSR2	ddPCR	3	0
Patient 109	11	ASXL1	ddPCR	11	0
Patient 2	4	Inversion 3	PCR	4	0
Patient 110	15	STAG2	Sanger	15	0
Totals:	79			78	1 WT, 1 Preleukemic

E

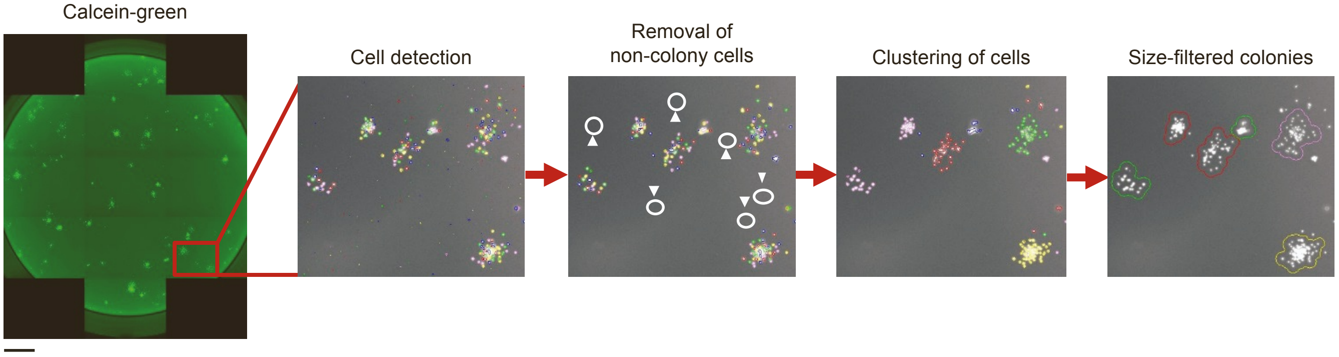

F

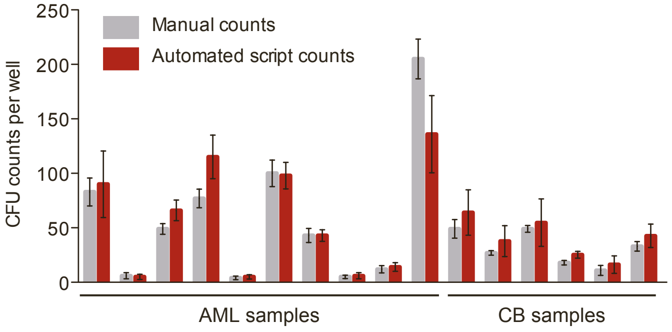

G

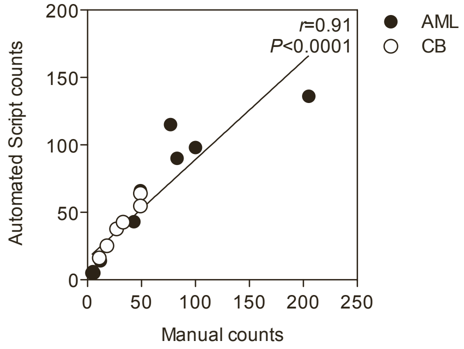

Figure S3. LPC assays are robust, reproducible, and can be automated. Related to Figure 2.
(A) Agarose gel electrophoresis showing PCR product specific for inversion 3 breakpoint region identified for Patient #2. DNA was extracted from bulk mononuclear cells or plucked colonies. (B) Summary of patient-specific mutation detection in plucked colonies, across 12 independent AML patients. For Patient #20, all colonies were counted as mutant due to the detection of at least one patient-specific mutation. (C) LPC frequencies in matched BM and PB samples collected from the same patients (collected <48 hours apart at diagnosis; n=9 patients). (D) Xenograft outcomes in matched BM and PB samples collected from the same patients (collected <48 hours apart at diagnosis; n=8 patients). Xenotransplantation was performed identically between BM and PB for all matched pairs (mouse strain and injection route). (E) Automated colony detection in high-throughput CFU platform. Scale bar, 2mm. (F) Automated script counts vs. manual counts of CFUs (n=3 wells from each of n=10 AML samples and n=5 healthy cord blood samples). CB, healthy cord blood. (G) Correlation of Automated script counts vs. Manual counts of CFUs. CB, healthy cord blood.

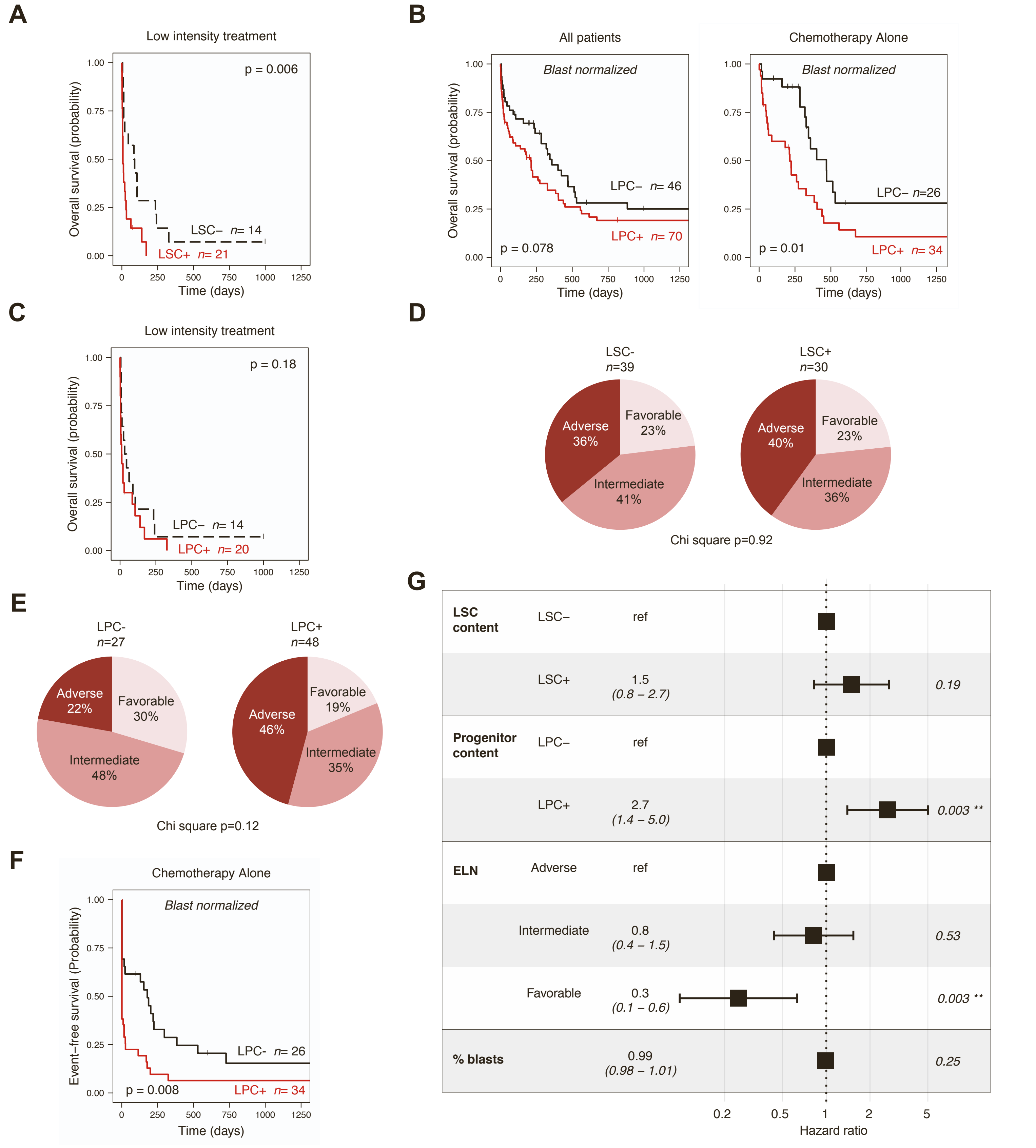

Figure S4. Functional LPC frequencies remain prognostic when normalized for leukemic blast content. Related to Figures 3 and 4.
(A) Kaplan-Meier estimates of Overall Survival in LSC- and LSC+ patient subsets (patients treated with low intensity therapy or palliative care only; *n*=35). (B) Kaplan-Meier estimates of Overall Survival in LPC- and LPC+ patient subsets, defined by LPC frequencies/10k leukemic blasts. Plots display all *n*=116 patients tested in colony forming LPC assays (left), or a subset of these patients treated with high intensity chemotherapy alone (palliative and HSCT-treated patients excluded; right). (C) Kaplan-Meier estimates of Overall Survival in LPC- and LPC+ patient subsets (patients treated with low intensity therapy or palliative care only; *n*=34). (D) Distribution of ELN genetic risk classes in LSC- vs. LSC+ patient subsets (*n*=69). (E) Distribution of ELN genetic risk classes in LPC- vs. LPC+ patient subsets (*n*=75). (F) Kaplan-Meier estimates of Event-free Survival in LPC- and LPC+ patient subsets, defined by LPC frequencies/10k leukemic blasts (patients treated with high intensity chemotherapy alone; *n*=60 patients). (G) Forest plot showing multivariate analysis of Event-free Survival in AML patients treated with high intensity chemotherapy alone (*n*=60 patients). Unadjusted HRs (squares) and 95% confidence intervals (horizontal lines) are shown.

Table S1. Clinical characteristics of patients evaluated by scRNAseq and/or Nanostring assays. Related to Figure 1.

ID#	Age	Sex	Genetics	AML engraftment	LPC freq/ 10k MNC	LSC17 classification	% CD34
1	56	M	46,XY[25]	N/A	N/A	N/A	N/A
2	48	M	45,XY,inv(3)(q21q26.2),-7[25]	Yes	12	High	70.1
3	84	M	Unknown karyotype, FLT3 ITD+NPM1-	No	45	High	7.9
4	71	F	Unknown karyotype, FLT3 ITD-NPM1-	No	0	Low	95
5	79	M	Complex	No	220	High	64.1
6	78	F	Complex	No	0	High	80.2
7	51	F	46,XX [25], FLT3 ITD+NPM1-	Yes	52	High	60
8	78	F	Complex	Yes	12	High	67.6
9	65	F	46,XX[20], FLT3 ITD+NPM1+	Yes	0	High	1.8
10	78	F	Unknown karyotype, FLT3 ITD+NPM1-	No	0	High	99
11	94	M	Unknown karyotype, FLT3 ITD-NPM1-	No	38	High	2
12	65	F	Unknown karyotype, FLT3 ITD+NPM1+	Yes	128	Low	6.8
13	61	M	46,XY,t(4;17)(q21;q21)[23]/46,XY[1], FLT3-ITD+NPM1+	Yes	18	High	59.1
14	56	F	50,XX,+8,+9,+14,+19[21]/46,XX[1]	No	34	Low	0.01
15	67	F	45,XX,-7[19]/46,XX[1]	Yes	45	High	70
16	73	F	Unknown karyotype, FLT3 ITD+NPM1+	Yes	1	Low	0.4
17	67	M	46,XY [3], FLT3 ITD-NPM1-	No	0	High	71.2
18	40	M	46,XY [15], FLT3-ITD+	Yes	28	High	37.3
19	62	M	Unknown karyotype, FLT3 ITD-NPM1+	Yes	0	Low	1
20	42	F	t(9;11), DNMT3A, ETV6, KRAS	Yes	23	Low	0.02
21	28	M	46,XY[20], FLT3 ITD-NPM1+	No	1	Low	0
22	70	F	46,XX [17], FLT3 ITD-NPM1-	Yes	1	High	95
23	65	M	CBFß/MYH11	No	65	High	80.6
24	44	F	46,XX [16], FLT3 ITD+NPM1+	Yes	2	N/A	57.8

Table S2. Hazard Ratios for Overall Survival in a preliminary cohort of 22 AML patients. Related to Figure 1.

Stemness measurement	HR	95% CI
%CD34	1.33	0.49-3.66
LSC17 score	1.39	0.49-3.95
LSC	1.27	0.46-3.55
CFU	7.13	1.55-32.8

Table S3. Serial xenotransplantation of AML patient samples. Related to Figure 1.

	1° xenograft			2° xenograft	
Patient ID	Leukemic chimerism	*n*	Experiment type	Leukemic chimerism	*n*
2	94.2 ± 2.5	5	mouse-to-mouse	94.2 ± 3	5
7	49.9 ± 22.2	4	mouse-to-mouse	64.9 ± 21.9	4
8	77.2 ± 4.6	6	Pooled*	0.4 ± 0.2	6
25	19 ± 4.4	8	Pooled	19.4 ± 3.7	6
26	38.1 ± 4.6	3	Pooled	22.4 ± 1.1	2
27	19.9 ± 8.5	3	Pooled	50.5 ± 8.2	3
28	70.2 ± 5.2	2	mouse-to-mouse	11.4 ±- 6.3	2
29	14 ± 6.9	4	mouse-to-mouse	1.3 ± 0.5	4
30	85.2 ± 6.2	4	mouse-to-mouse	0.1 ± 0.1	3

Chimerism values represent mean ± s.e.m.
*Transplanted at limiting dilution

Table S4. Primer sequences. Related to Figure 2 and STAR Methods.

Target	Application	Forward primer	Reverse primer	Probe 1	Probe 2
DNMT3A c.2645G>A	ddPCR	CCCAGGGTATTTGGTTTCCC	GAAGAGGTGGCGGATGACT	AACATGAGCCACTTG	ACATGAGCCGCTTGG
ETV6 c.331G>A	ddPCR	TGCTGCTCCGTAGATCGTTG	CCGGCTGTGTGTGTATAGAGT	TTCCAGGTAATGTGC	TTCCAGGTGATGTGC
TP53 c.842A>T	ddPCR	GAACAGCTTTGAGGTGCGTG	TGTCCTGCTTGCTTACCTCG	CCTGGGAGAGTCC	TCCTGGGAGAGACC
GRM8 c.10G>A	ddPCR	GTAGCCTCCAGAAGGTGCAG	CCAGTAGAACTTGGCGGTCA	TGGTATGCAAGGGAA	TGGTATGCGAGGGAA
NRAS c.38G>T	ddPCR	ACAGGTTCTTGCTGGTGTGAA	TGGATTGTCAGTGCGCTTTTC	AGCAGGTG**G**TGTTGG	AGCAGGTG**T**TGTTGG
TET2 c.1630C>T	ddPCR	TGCCAGCAGTTGATGAGAAACA	TCCGCTTGGTGAAAACGAGG	ATTCTGAAGGGT**C**GAGAC	ATTCTGAAGGGT**T**GAGAC
IDH2 c.419G>A	ddPCR	TGGGCTCCCGGAAGACA	AAGATGTGGAAAAGTCCCAATGGA	CTATCCGGAACATCC	ACTATCCAGAACATCC
ZRSR2 c.810_811insAT	ddPCR	GTCAGCTGCAATTTGGAACCT	GGAAGACATCCACAAGCAGAATACT	CTGGTACTGAACATATACATT	TGGTACTGAACATAATTACATT
ASXL1 c.2725A>T	ddPCR	CGTTTCTAACAGTTCTTTGCATTGGA	TGTGTTCTCTGGATTCTGGTTTGG	TGAGGTAGTGAAACAGC	ATGAGGTAGTGTAACAGC
STAG2:c.2924+3A>C	conventional PCR	GCTCGACGTTTTGCTTTAACT	AGATAGCACTGTAACTGGTTCTT	N/A	N/A
Inversion 3	conventional PCR	CCTCTGAGCAACTTTGCTTTC	GGCTGGCAGAAAATCTTCAC	N/A	N/A

Table S5. Multivariate analysis of Overall Survival in AML patients treated with high intensity chemotherapy alone (*n*=60 patients), classified based on ELN 2022 risk status. Related to Figure 4.

Variable		Hazard ratio	P value
LSC content	LSC-	reference	
	LSC+	0.98 (0.49 - 1.96)	0.96
LPC content	LPC-	reference	
	LPC+	2.63 (1.21 - 5.72)	0.015*
ELN 2022	Adverse	reference	
	Intermediate	1.45 (0.70 - 2.99)	0.31
	Favorable	0.28 (0.08 - 0.97)	0.04*
% blasts		1.02 (0.995 - 1.05)	0.1

*statistically significant

Table S6. Multivariate analysis of Event-free Survival in AML patients treated with high intensity chemotherapy alone (*n*=60 patients), classified based on ELN 2022 risk status. Related to Figure 4.

Variable		Hazard ratio	P value
LSC content	LSC-	reference	
	LSC+	1.52 (0.81 - 2.83)	0.19
LPC content	LPC-	reference	
	LPC+	2.67 (1.29 - 5.49)	0.008*
ELN 2022	Adverse	reference	
	Intermediate	1.02 (0.49 - 2.08)	0.97
	Favorable	0.22 (0.08 - 0.64)	0.006*
% blasts		0.99 (0.98 - 1.01)	0.26

*statistically significant

Supplemental References

S1. Eppert K, Takenaka K, Lechman ER, Waldron L, Nilsson B, van Galen P, Metzeler KH, Poeppl A, Ling V, Beyene J, et al. (2011). Stem cell gene expression programs influence clinical outcome in human leukemia. Nat Med 17, 1086-1093. 20110828.

S2. Gal H, Amariglio N, Trakhtenbrot L, Jacob-Hirsh J, Margalit O, Avigdor A, Nagler A, Tavor S, Ein-Dor L, Lapidot T, et al. (2006). Gene expression profiles of AML derived stem cells; similarity to hematopoietic stem cells. Leukemia 20, 2147-2154. 20061012.

S3. Jaatinen T, Hemmoranta H, Hautaniemi S, Niemi J, Nicorici D, Laine J, Yli-Harja O and Partanen J. (2006). Global gene expression profile of human cord blood-derived CD133+ cells. Stem Cells 24, 631-641. 20051006.

S4. Aslostovar L, Boyd AL, Benoit YD, Di Lu J, Garcia Rodriguez JL, Nakanishi M, Porras DP, Reid JC, Mitchell RR, Leber B, et al. (2021). Abnormal dopamine receptor signaling allows selective therapeutic targeting of neoplastic progenitors in AML patients. Cell Rep Med 2, 100202. 20210216.

S5. Lee JH, Salci KR, Reid JC, Orlando L, Tanasijevic B, Shapovalova Z and Bhatia M. (2017). Brief Report: Human Acute Myeloid Leukemia Reprogramming to Pluripotency Is a Rare Event and Selects for Patient Hematopoietic Cells Devoid of Leukemic Mutations. Stem Cells 35, 2095-2102. 20170731.
